# Supplementary material for: A synthetic antibiotic class with a deeply-optimized design for overcoming bacterial resistance
Source: Nat Commun. 2024 Jul 18;15:6040. doi: 10.1038/s41467-024-50453-3 (PMC11255307; doi:10.1038/s41467-024-50453-3)

Supplementary Information for

A synthetic antibiotic class with a deeply-optimized design for overcoming bacterial resistance

Jin Feng[a], Youle Zheng[b], Wanqing Ma[b], Defeng Weng[b], Dapeng Peng[a,b], Yindi Xu[c], Zhifang Wang[c], Xu Wang[a,b*]

[a] National Reference Laboratory of Veterinary Drug Residues (HZAU) and MAO Key Laboratory for Detection of Veterinary Drug Residues, Huazhong Agricultural University, Wuhan, Hubei 430070, China.

[b] MAO Laboratory for Risk Assessment of Quality and Safety of Livestock and Poultry Products, Huazhong Agricultural University, Wuhan, Hubei 430070, China.

[c] Institute of Animal Husbandry and Veterinary Research, Henan Academy of Agricultural Sciences, Zhengzhou, Henan 450002, China.

Corresponding authors:

*Prof. Dr. Xu Wang, PhD,

National Reference Laboratory of Veterinary Drug Residues (HZAU) and

MAO Key Laboratory for Detection of Veterinary Drug Residues,

Wuhan, Hubei 430070, China.

E-mail address: wangxu@mail.hzau.edu.cn

1. Supplementary Figures

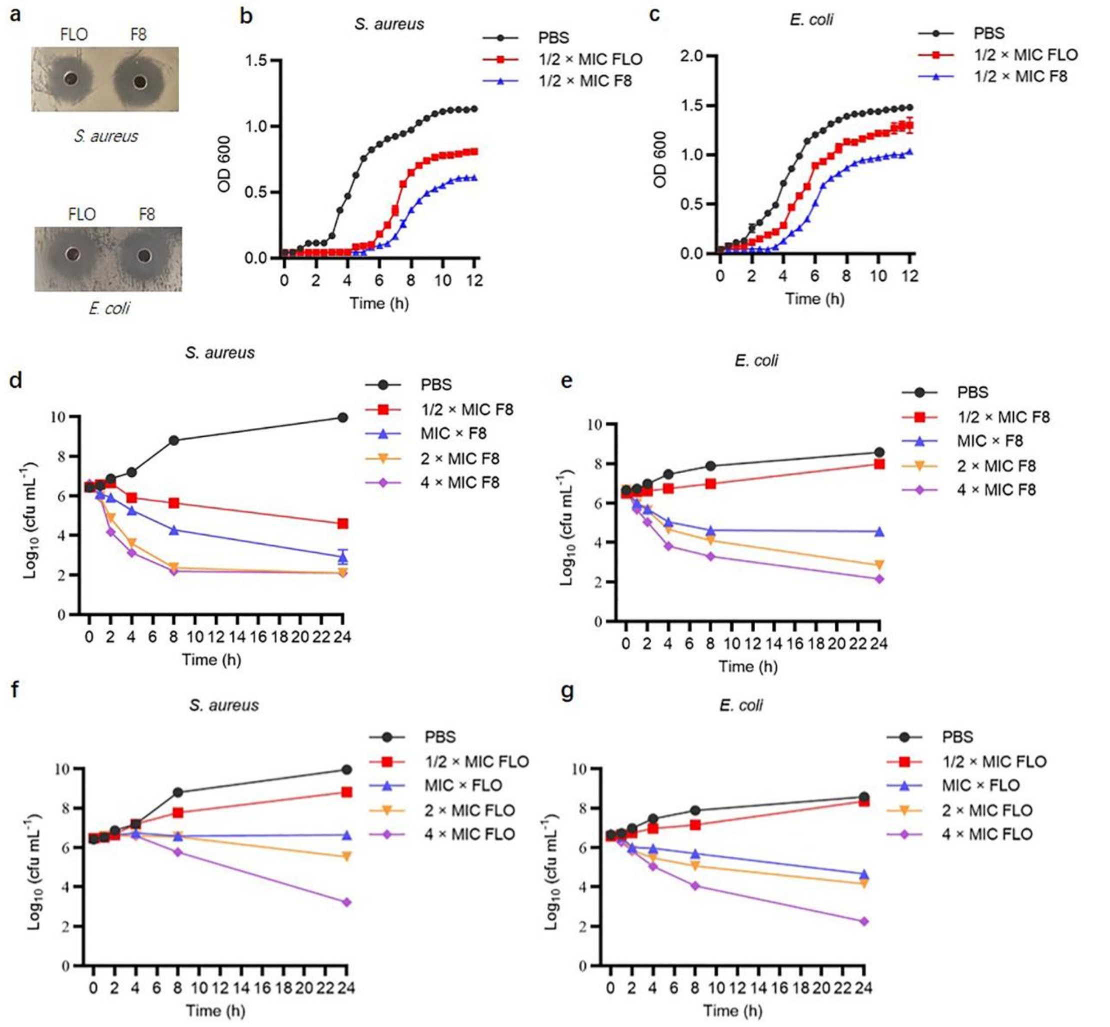

Supplementary Fig. 1. Antimicrobial activity of F8 *in vitro*. a, Agar diffusion assay of F8 against *S. aureus* and *E. coli*. **b, c,** Growth curves of *S. aureus* and *E. coli* cultured with F8 at 1/2×MIC concentration or positive control FLO. **d, e,** Kill-time curves of *S. aureus* and *E. coli* with different concentrations of F8. **f, g,** Kill-time curves of *S. aureus* and *E. coli* with different concentrations of FLO. Source data are provided as a Source Data file.

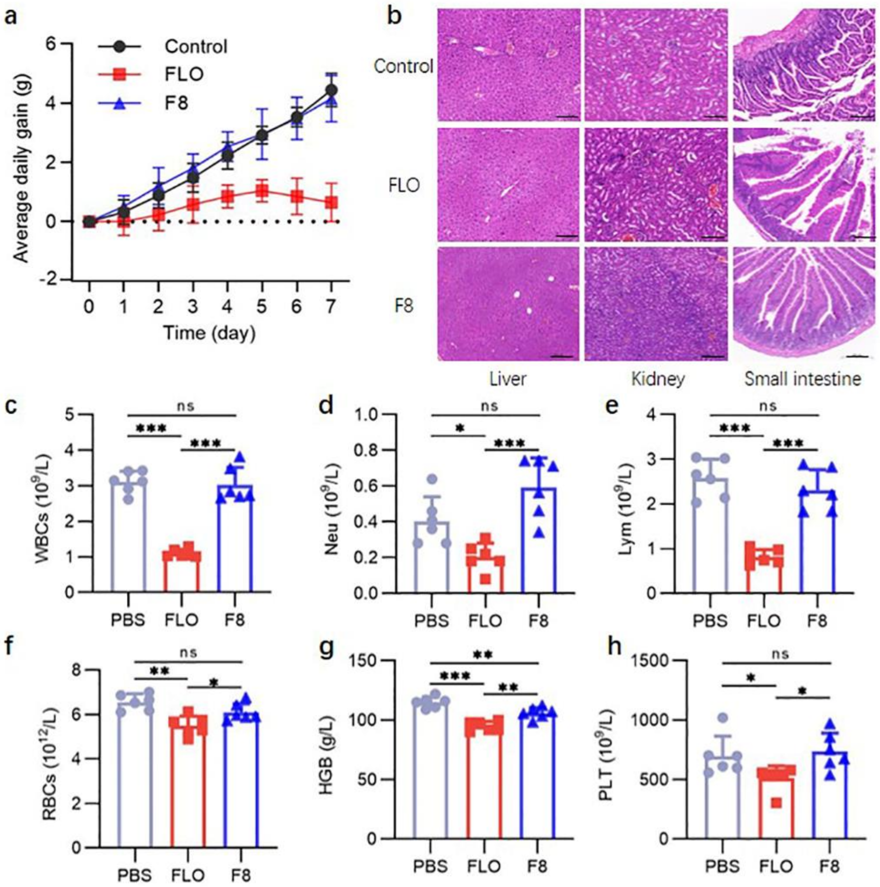

Supplementary Fig. 2. Safety evaluation of F8. a, Record of mouse body weight after treatment with F8 at a dose of 1,500 mg/kg or positive control FLO (n = 6). **b,** Histopathological assessment of spleen, thymus, and bone marrow in mice after treatment with F8 or positive control FLO at a dose of 1500 mg/kg (n=6). **c–h,** Count of white blood cells (WBCs), neutrophils (Neu), lymphocytes (Lym), red blood cells (RBCs), haemoglobin (HGB), and platelets (PLT) in different groups. (n=6). Statistics: two tailed t test. Mean with SD. c (p<0.0001 (FLO-PBS), p=0.7414 (F8-PBS), p=<0.0001 (F8-FLO)). d (p=0.0100 (FLO-PBS), p=0.0594 (F8-PBS), p=0.0004 (F8-FLO)). e (p<0.0001 (FLO-PBS), p=0.3204 (F8-PBS), p<0.0001 (F8-FLO)). f (p=0.0010 (FLO-PBS), p=0.0615 (F8-PBS), p=0.0332 (F8-FLO)). g (p<0.0001 (FLO-PBS), p=0.0067 (F8-PBS), p=0.0022 (F8-FLO)). h (p=0.0421 (FLO-PBS), p=0.6997 (F8-PBS), p=0.0149 (F8-FLO)). ns, not significant, *, $p < 0.05$, **, $p < 0.01$, ***, $p < 0.001$. Source data are provided as a Source Data file.

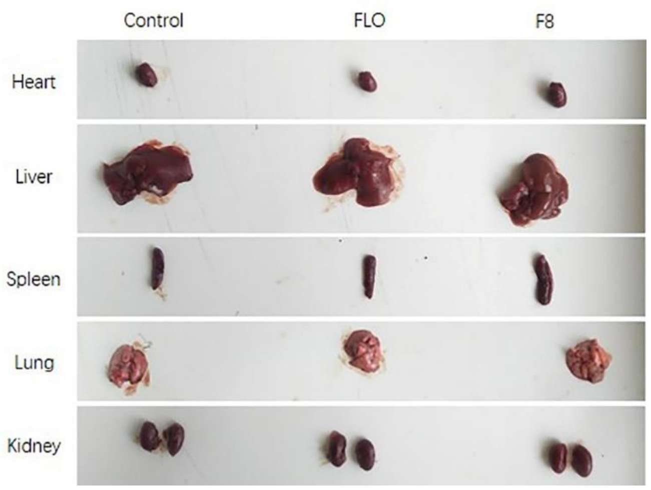

Supplementary Fig. 3. Acute toxicity of F8 and FLO in mice. Gross autopsy of heart, liver, spleen, lungs, and kidneys.

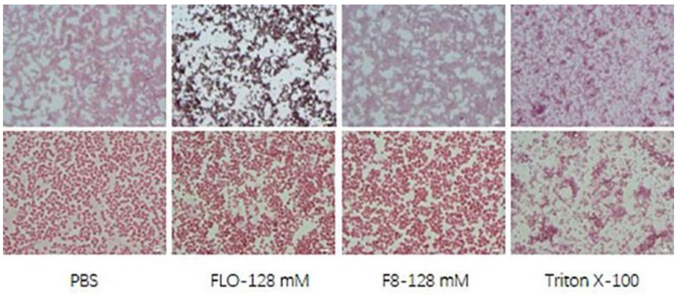

Supplementary Fig. 4. Hemolytic activity of F8 and FLO. Blood cell smear of sheep red blood cells by different concentrations of F8 or positive control FLO (0 – 256 μM) (n=6).

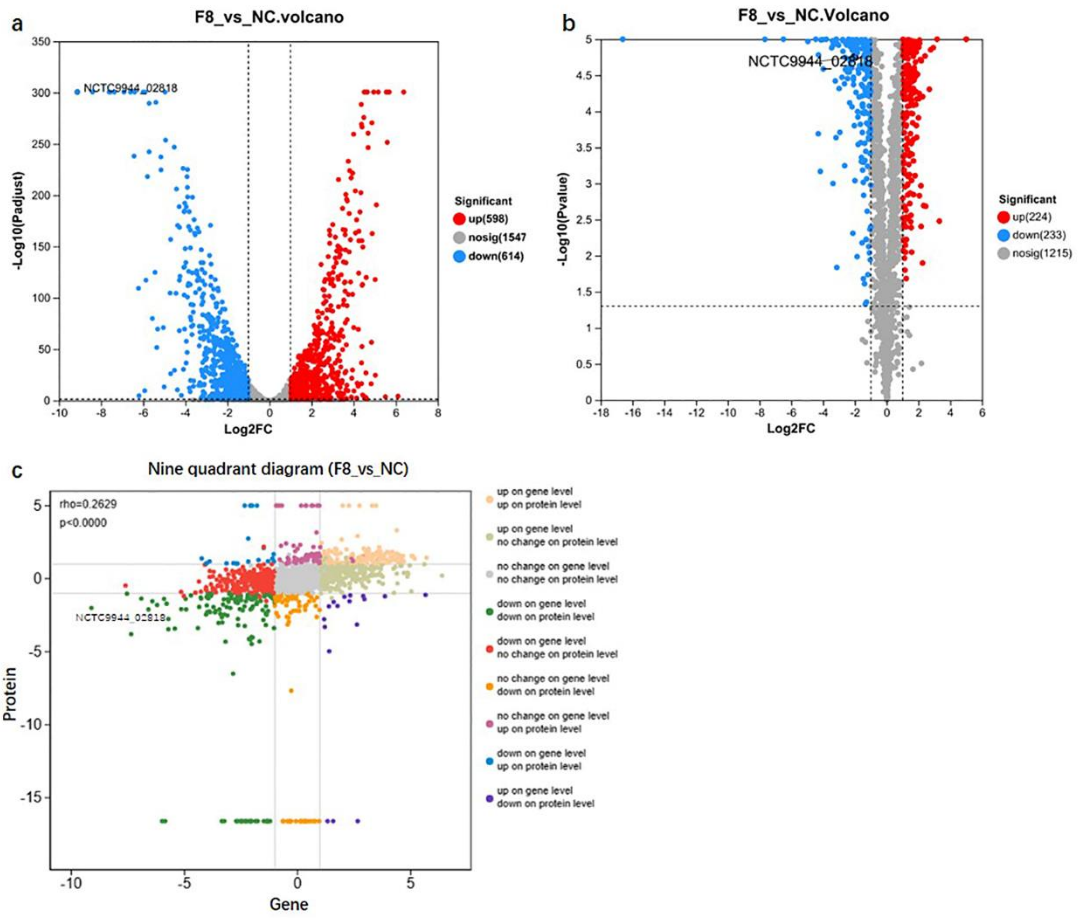

Supplementary Fig. 5. Mult-omics analysis of F8 treatment on *S. aureus* ATCC 29213. a, Volcano plot of differentially-expressed genes (DEGs). **b,** Volcano plot of differentially-expressed proteins (DEPs). **c,** Correlation analysis between DEGs and DEPs using a nine-quadrant diagram.

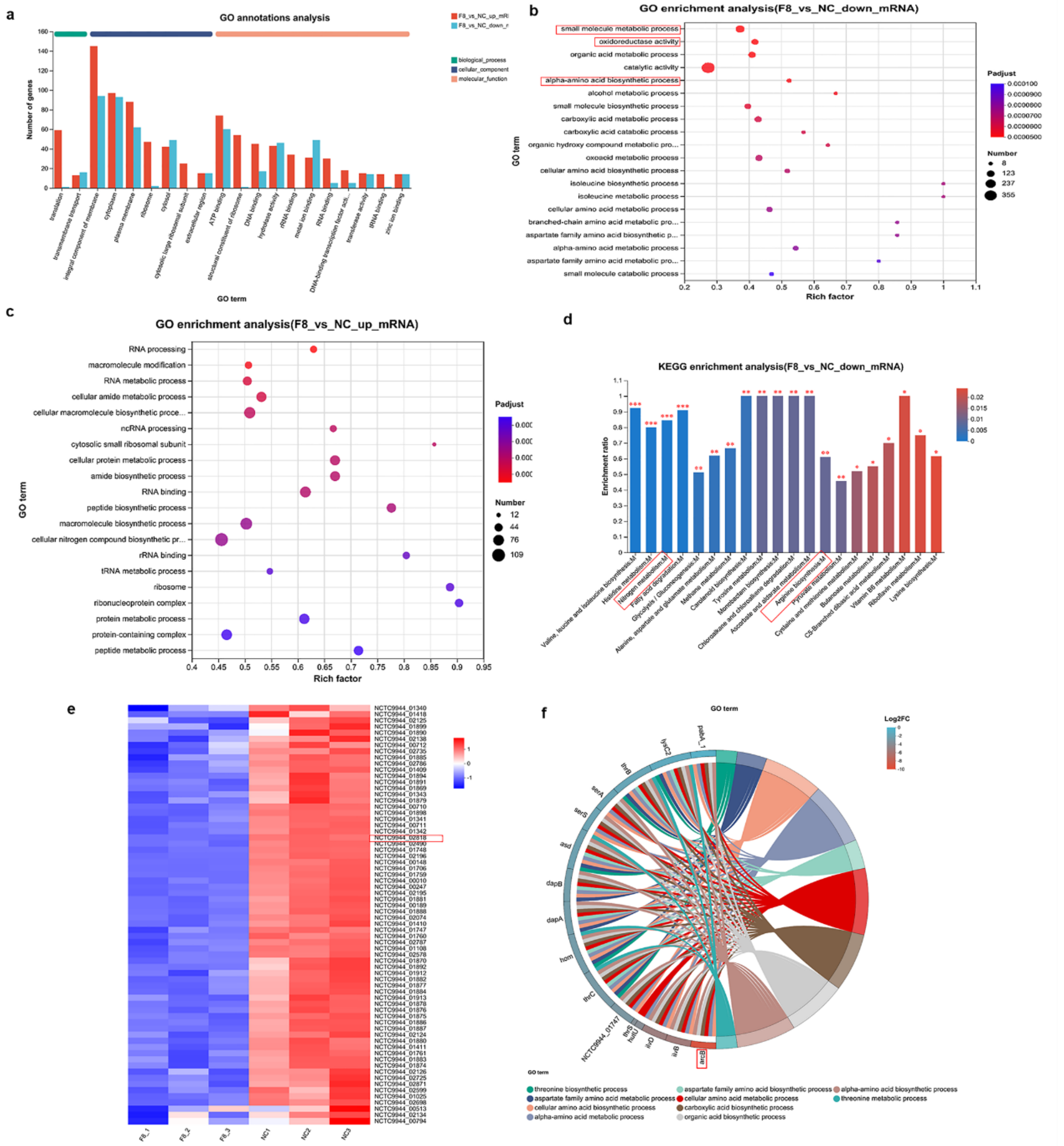

Supplementary Fig. 6. Transcriptomic analysis of F8 treatment on *S. aureus* ATCC 29213. a, GO annotation analysis. **b, c,** GO enrichment analysis. **d,** KEGG enrichment analysis. The x-axis and y-axis represent expression changes and the corresponding statistical significance, respectively. **e,** Clustering heatmap of some important DEGs. **f,** GO functional enrichment chord diagram.

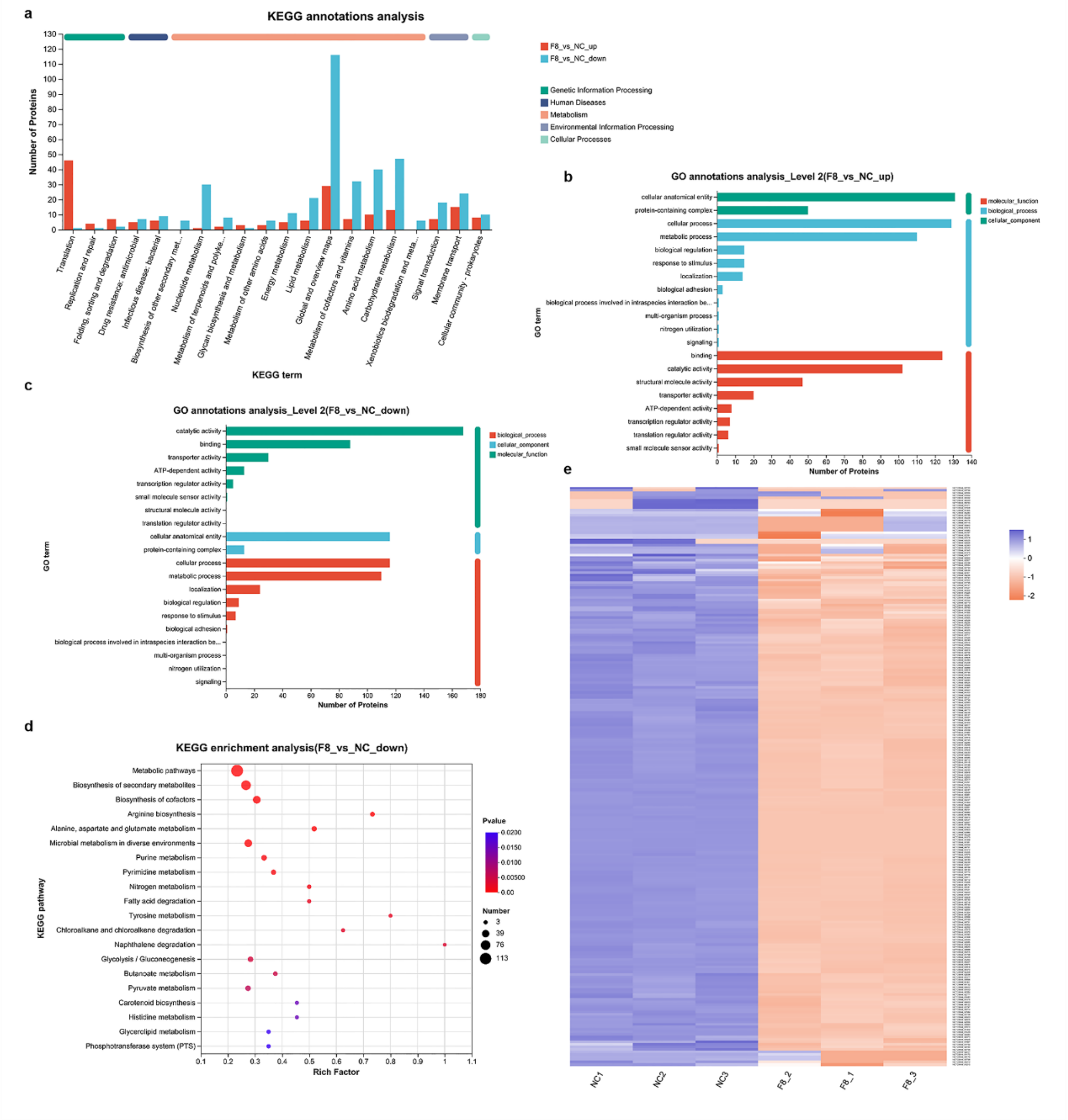

Supplementary Fig. 7. Proteomic analysis of F8 treatment on *S. aureus* ATCC 29213. a, KEGG annotation analysis. **b, c,** GO enrichment analysis. **d,** KEGG enrichment analysis. **e,** Clustering heatmap of some important DEPs.

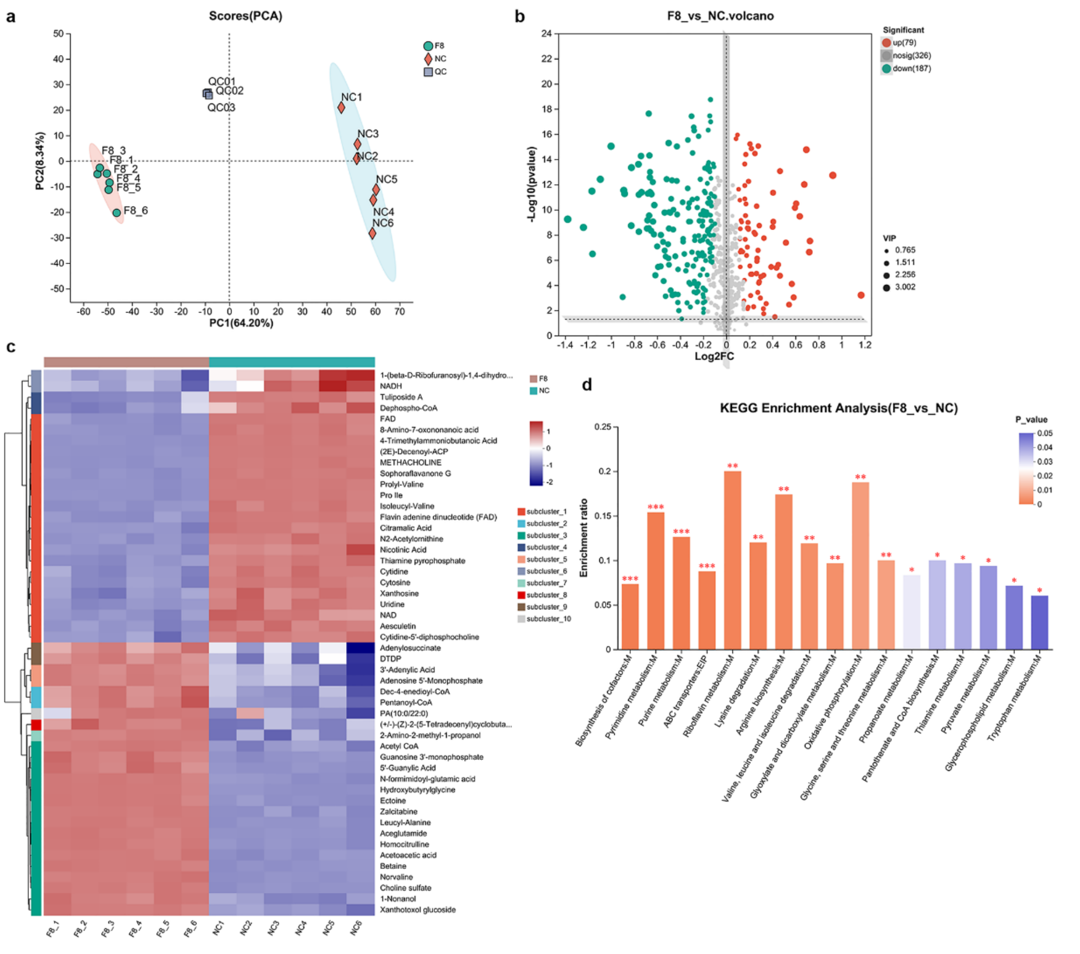

Supplementary Fig. 8. Metabolomic analysis of F8 treatment on *S. aureus* ATCC 29213. a, Principal Component Analysis (PCA). **b,** Volcano plot of differentially-expressed metabolites. **c,** Clustering heatmap of metabolic profiles. **d,** KEGG enrichment analysis. The x-axis and y-axis represent expression changes and the corresponding statistical significance, respectively.

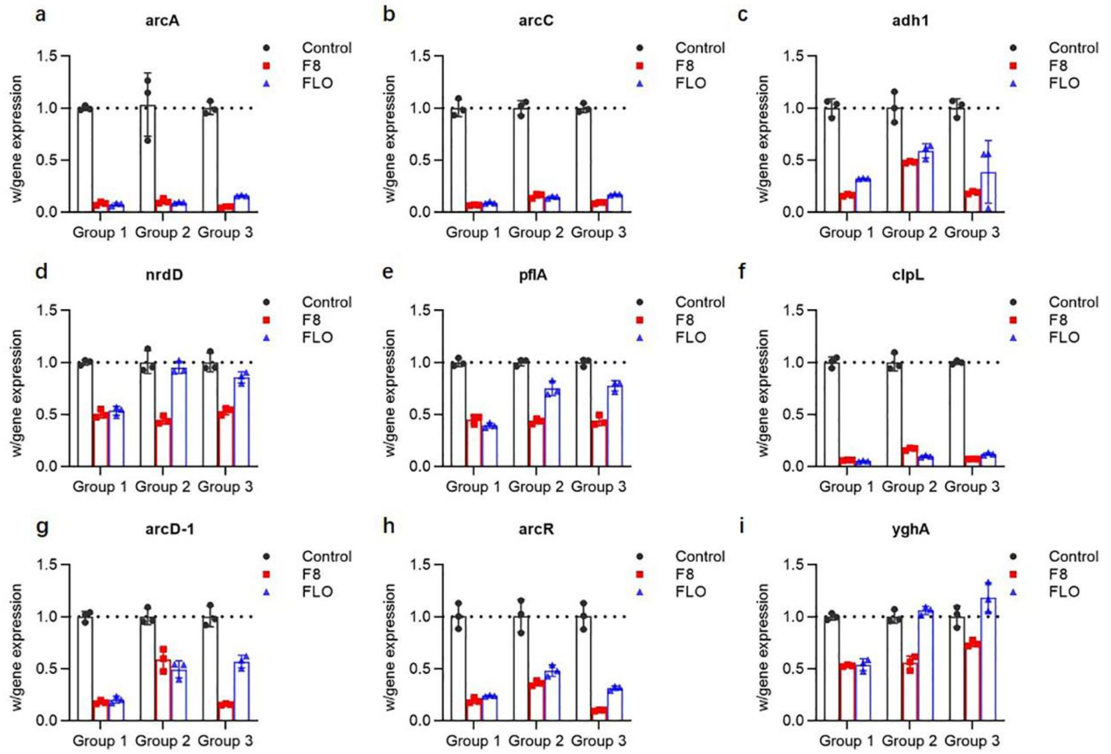

Supplementary Fig. 9. Differentially expressed genes of *S. aureus* with F8. a–i, Using RT-qPCR to detect the differentially expressed genes in *S. aureus* after treatment with F8 or positive control FLO. Groups 1-3 represent three sets of transcriptomic samples, respectively. (n=3). Mean with SD. Source data are provided as a Source Data file.

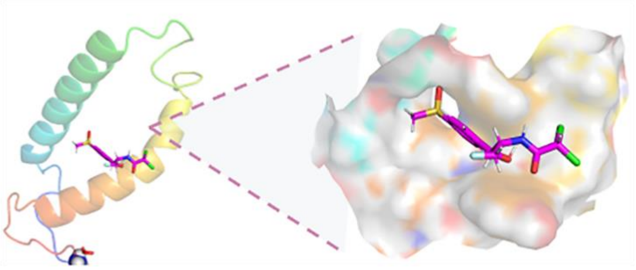

Supplementary Fig. 10. The binding of arcB and FLO. The simulated image of the 4 Å drug binding domain (DBD) of FLO in arcB (PDB: 2ksd) generated by PyMOL shows a magenta color.

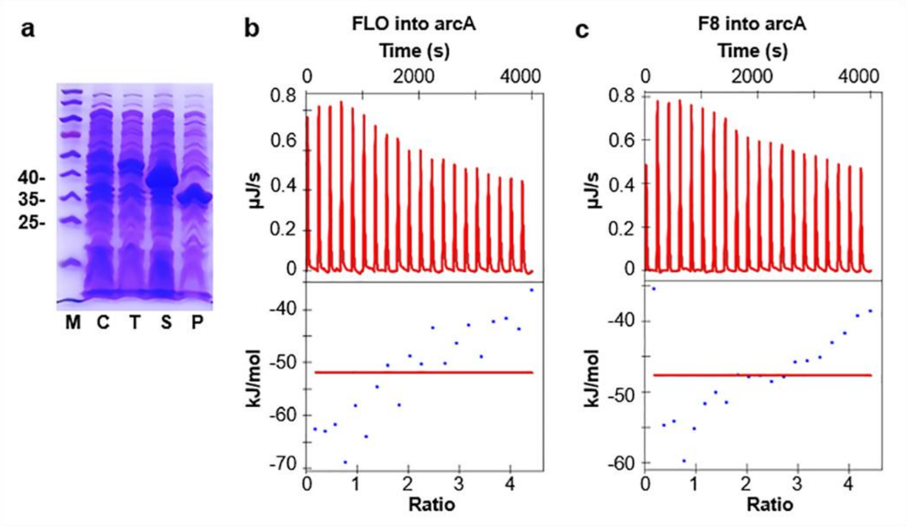

Supplementary Fig. 11. The binding of arcA with F8 or FLO. a, Expression of arcA, in SDS-PAGE analysis, the target protein expressed in total protein (T), soluble (S), and insoluble (P) fractions of the lysate compared with negative control (C, empty pET28a vector). The expected size of arcB is 46.93 kDa. 3 times each experiment was repeated independently with similar results. **b**, **c**, ITC detection of ligand-target binding affinity.

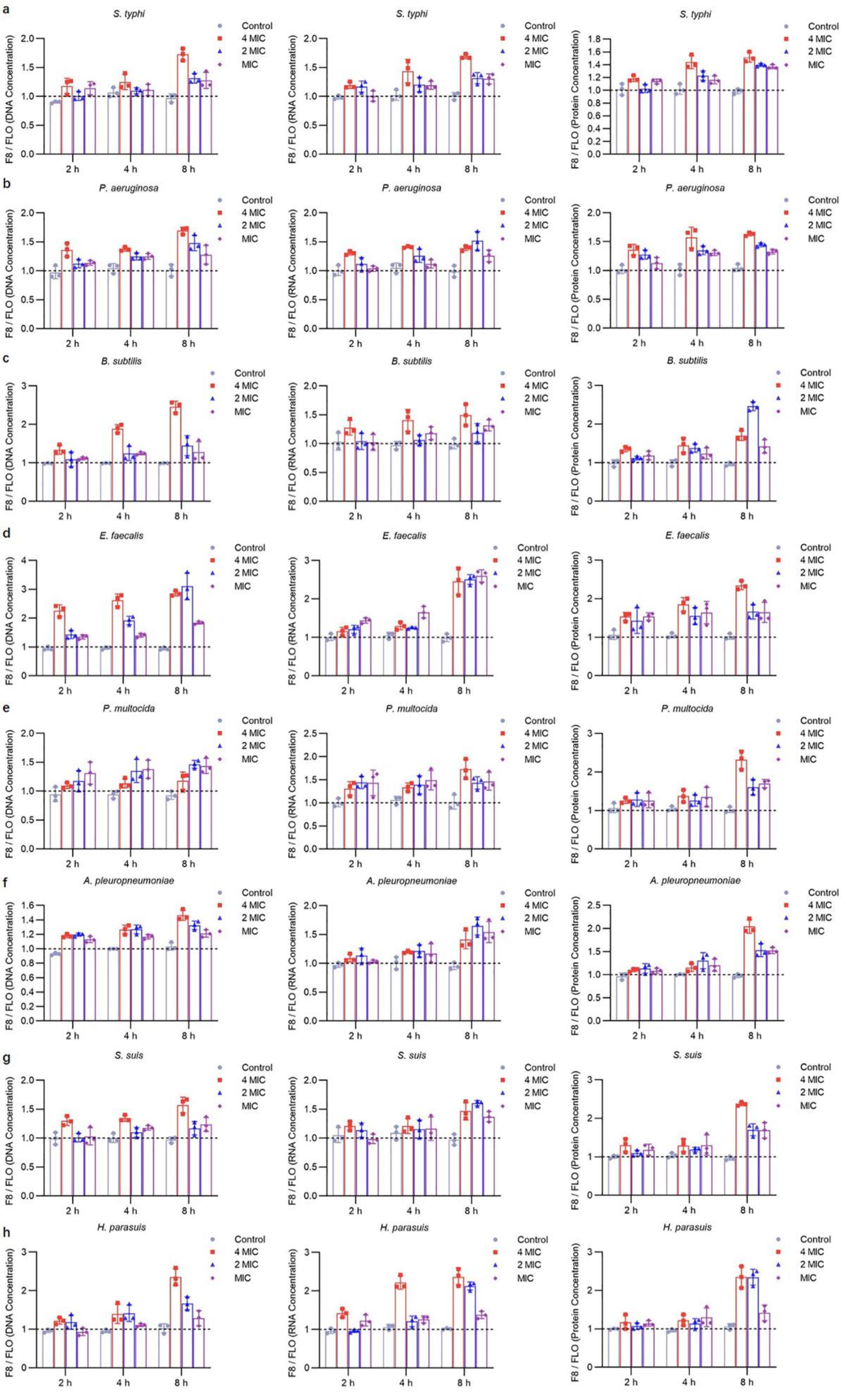

Supplementary Fig. 12. Effect of F8 on bacterial content. Changes in extracellular

DNA, RNA, and protein concentrations at different time points after treatment with different concentrations of F8 (4×MIC, 2×MIC, and MIC). **a**, *S. typhi*. **b**, *P. aeruginosa*. **c**, *B. subtilis*. **d**, *E. faecalis*. **e**, *P. multocida*. **f**, *A. pleuropneumoniae*. **g**, *S. suis*. **h**, *H. parasuis*. (n=3). Mean with SD. Source data are provided as a Source Data file.

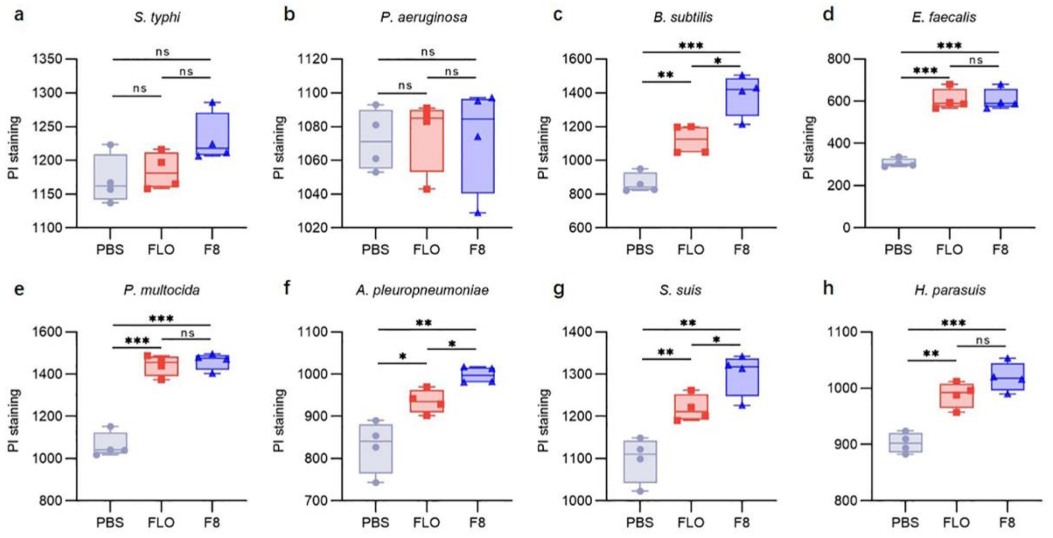

Supplementary Fig. 13. Effect of F8 on bacterial cytoplasmic membrane permeability. Increased membrane permeability in bacteria after treatment with 4×MIC of F8. Membrane permeability measured by propidium iodide (PI), with excitation/emission wavelength at 535 nm/615 nm. **a**, *S. typhi*. **b**, *P. aeruginosa*. **c**, *B. subtilis*. **d**, *E. faecalis*. **e**, *P. multocida*. **f**, *A. pleuropneumoniae*. **g**, *S. suis*. **h**, *H. parasuis*. (n=4). Statistics: two tailed t test. In boxplots the lower hinge represents 25% quantile, upper hinge 75% quantile, and center line the median. a (p=0.5911 (FLO-PBS), p=0.0588 (F8-PBS), p=0.0828 (F8-FLO)). b (p=7905 (FLO-PBS), p=9267 (F8-PBS), p=0.9111 (F8-FLO)). c (p=0.0026 (FLO-PBS), p=0.0003 (F8-PBS), p=0.0127 (F8-FLO)). d (p<0.0001 (FLO-PBS), p<0.0001 (F8-PBS), p>0.9999 (F8-FLO)). e (p<0.0001 (FLO-PBS), p<0.0001 (F8-PBS), p=0.5770 (F8-FLO)). f (p=0.0205 (FLO-PBS), p=0.0020 (F8-PBS), p=0.0108 (F8-FLO)). g (p=0.0087 (FLO-PBS), p=0.0016 (F8-PBS), p=0.0347 (F8-FLO)). h (p=0.0011 (FLO-PBS), p=0.0003 (F8-PBS), p=0.1191 (F8-FLO)). ns, not significant; *, $p < 0.05$; **, $p < 0.01$; ***, $p < 0.001$. Source data are provided as a Source Data file.

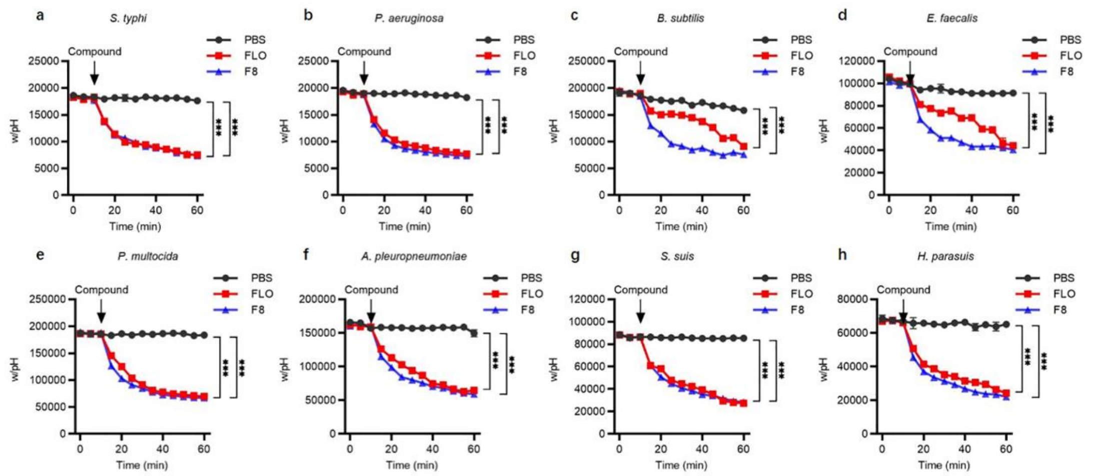

Supplementary Fig. 14. Effect of F8 on bacterial proton kinetic parameters. Detection of ΔpH in bacteria after treatment with 4×MIC of F8. Measured using the fluorescent probe, BCECF-AM, with excitation/emission wavelength at 488 nm/535 nm. **a**, *S. typhi*. **b**, *P. aeruginosa*. **c**, *B. subtilis*. **d**, *E. faecalis*. **e**, *P. multocida*. **f**, *A. pleuropneumoniae*. **g**, *S. suis*. **h**, *H. parasuis*. (n=3). Statistics: two tailed t test. a (p<0.0001 (FLO-PBS), p<0.0001 (F8-PBS)). b (p<0.0001 (FLO-PBS), p<0.0001 (F8-PBS)). c (p=0.0006 (FLO-PBS), p<0.0001 (F8-PBS)). d (p=0.0006 (FLO-PBS), p<0.0001 (F8-PBS)). e (p=0.0001 (FLO-PBS), p<0.0001 (F8-PBS)). f (p=0.0001 (FLO-PBS), p<0.0001 (F8-PBS)). g (p=0.0001 (FLO-PBS), p<0.0001 (F8-PBS)). h (p<0.0001 (FLO-PBS), p<0.0001 (F8-PBS)). ns, not significant; *, $p < 0.05$; **, $p < 0.01$; ***, $p < 0.001$. Source data are provided as a Source Data file.

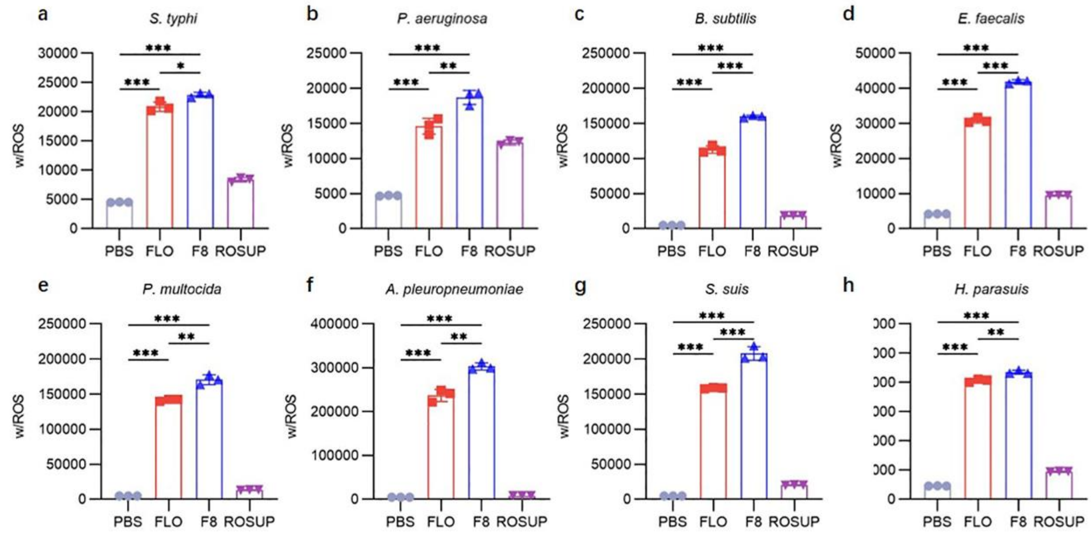

Supplementary Fig. 15. Effect of F8 on bacterial ROS. Changes in intracellular ROS levels in bacteria after treatment with 4×MIC of F8. Using ROSUP as a positive control. **a**, *S. typhi*. **b**, *P. aeruginosa*. **c**, *B. subtilis*. **d**, *E. faecalis*. **e**, *P. multocida*. **f**, *A. pleuropneumoniae*. **g**, *S. suis*. **h**, *H. parasuis*. (n=3). Statistics: two tailed t test. a (p<0.0001 (FLO-PBS), p<0.0001 (F8-PBS), p=0.0185 (F8-FLO)). b (p=0.0001 (FLO-PBS), p<0.0001 (F8-PBS), p=0.0095 (F8-FLO)). c (p<0.0001 (FLO-PBS), p<0.0001 (F8-PBS), p=0.0001 (F8-FLO)). d (p<0.0001 (FLO-PBS), p<0.0001 (F8-PBS), p<0.0001 (F8-FLO)). e (p<0.0001 (FLO-PBS), p<0.0001 (F8-PBS), p=0.0023 (F8-FLO)). f (p<0.0001 (FLO-PBS), p<0.0001 (F8-PBS), p=0.0021 (F8-FLO)). g (p<0.0001 (FLO-PBS), p<0.0001 (F8-PBS), p=0.0009 (F8-FLO)). h (p<0.0001 (FLO-PBS), p<0.0001 (F8-PBS), p=0.0039 (F8-FLO)). ns, not significant; *, $p < 0.05$; **, $p < 0.01$; ***, $p < 0.001$. Source data are provided as a Source Data file.

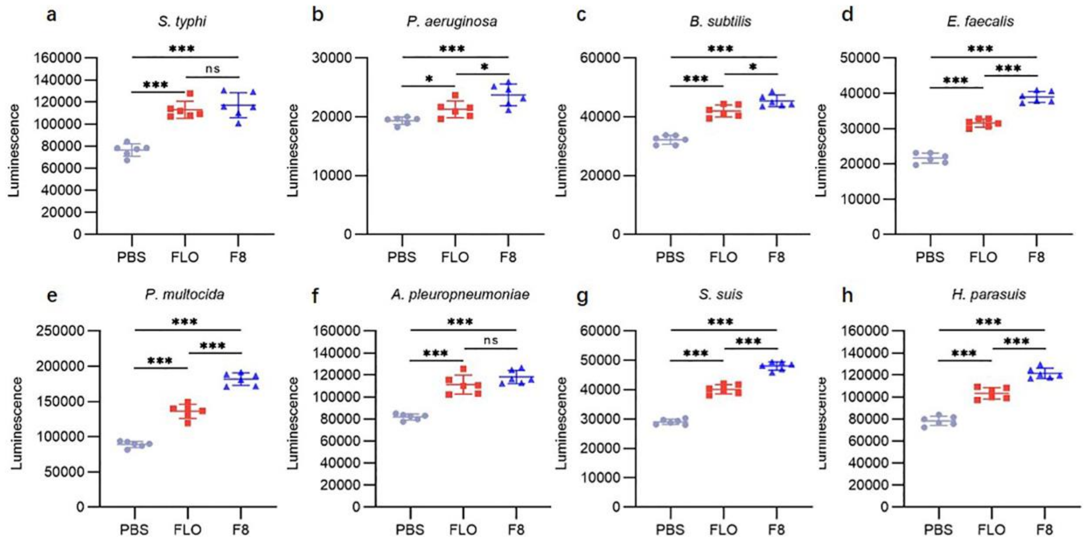

Supplementary Fig. 16. Effect of F8 on bacterial ATP levels. Changes in intracellular ATP levels in bacteria after treatment with 4×MIC of F8. **a**, *S. typhi*. **b**, *P. aeruginosa*. **c**, *B. subtilis*. **d**, *E. faecalis*. **e**, *P. multocida*. **f**, *A. pleuropneumoniae*. **g**, *S. suis*. **h**, *H. parasuis*. (n=6). Statistics: two tailed t test. a (p<0.0001 (FLO-PBS), p<0.0001 (F8-PBS), p=0.4780 (F8-FLO)). b (p=0.0121 (FLO-PBS), p=0.0003 (F8-PBS), p=0.0264 (F8-FLO)). c (p<0.0001 (FLO-PBS), p<0.0001 (F8-PBS), p=0.0151 (F8-FLO)). d (p<0.0001 (FLO-PBS), p<0.0001 (F8-PBS), p<0.0001 (F8-FLO)). e (p<0.0001 (FLO-PBS), p<0.0001 (F8-PBS), p<0.0001 (F8-FLO)). f (p<0.0001 (FLO-PBS), p<0.0001 (F8-PBS), p=0.1379 (F8-FLO)). g (p<0.0001 (FLO-PBS), p<0.0001 (F8-PBS), p<0.0001 (F8-FLO)). h (p<0.0001 (FLO-PBS), p<0.0001 (F8-PBS), p<0.0001 (F8-FLO)). ns, not significant; *, $p < 0.05$; **, $p < 0.01$; ***, $p < 0.001$. Source data are provided as a Source Data file.

2. Supplementary Tables

Supplementary Table 1. SYBYL simulation docking.

Name	Mol Wt	Total Score	Crash	Polar
F14	559	7.6589	-2.6455	5.0034
F8	497	6.1738	-1.9481	4.0442
F3	425	6.0496	-2.1376	3.6280
F1	453	5.9889	-1.1184	4.1346
F15	603	5.7043	-3.3284	2.1640
F4	440	5.6470	-0.9994	4.2054
F11	513	5.6375	-0.9141	2.3341
F2	514	5.4920	-1.0737	4.4195
F13	485	5.3018	-1.6204	3.4649
F7	475	5.2215	-2.1318	4.3768
FLO	358	5.1467	-2.2698	4.9464
F6	468	5.0195	-1.1588	3.3045

a. SYBYL simulation docking between 12 molecular structures and the PTC region of the 50s subunit. b. FLO, florfenicol.

Supplementary Table 2. General chemistry information.

Name	Fraction (CAS number)	Source of fraction[a]	Eluant (hexane/EtOAc)	Yield of product (%)
F8	3-(1-Piperidinyl)propanoic acid (CAS: 26371-07-3)	Add 20 mL of N,N-Dimethylformamide (DMF), 2 g of 3-(1-Piperidinyl)propanoic acid (CAS: 26371-07-3) until completely dissolved, followed by 1.5 g of 4-Dimethylaminopyridine (DMAP), 3 g of dichloroethane (EDC), and 3 g of FLO. Stir magnetically at room temperature for 24 h and take samples for reaction detection	2/1	41
F1	3-Oxocyclobutanecarboxylic acid (CAS: 23761-23-1)	same as above	2/1	46
F2	Folcisteine (5025-82-1)	same as above	3/2	62
F3	Cyclopropanecarboxylic acid (1759-53-1)	same as above	2/1	51
F4	Cyclo-butyl formic acid (3721-95-7)	same as above	2/1	42
F6	2-(1-Pyrrolidyl)acetic Acid (37386-15-5)	same as above	2/1	39
F7	3,3-Difluorocyclobutanecarboxylic acid (107496-54-8)	same as above	2/1	39
F11	6-Quinolinecarboxylic acid (10349-57-2)	same as above	2/1	45
F13	Morpholin-4-yl-acetic acid (3235-69-6)	same as above	2/1	41
F14	3-phthalimidopropionic acid (3339-73-9)	same as above	2/1	52
F15	N-Cbz- Piperidine-3-carboxylic acid (78190-11-1)	same as above	2/1	42

a. Unless otherwise noted, all materials were obtained from commercial suppliers and used without further purification.

Supplementary Table 3. RT-qPCR assays primers.

Gene name	Primer sequence5’-3’
16sJ-F	CGTGCTACAATGGACAATACAAA
16sJ-R	ATCTACGATTACTAGCGATTCCA
arcB-F	GCGAGTCAATATGGTGGTTCAGTC
arcB-R	CACCCATCGATACCCAAACATCTG
arcA-F	GGAGAGCACGACGACGAGAATC
arcA-R	AAAACAAGTTCATCGCCGCCTTC
arcC-F	TGCGGTGGTGGCGGTATTC
arcC-R	TGCTTCAATCAGCGTTGCTAATTTC
adh1-F	AGATGGTGCAATGGCTGAACAAG
adh1-R	TAGAAGACGCTGCTGCTGGATC
nrdD-F	CAAACTGGGAAACATCTCAAGAAGC
nrdD-R	TTCACTCGGCGTACTGTAAATACTG
pflA-F	TGGCGGTGTAACAGTCAGTGG
pflA-R	CAGCCGATGTGTCTAAGCAAGTG
clpL-F	CATTAGAAGCTGGAACGCAATATCG
clpL-R	TCCTGTGGCACCTGAACCG
arcD-1-F	TCTCGGTTGTAATTGCCTCTAATGC
arcD-1-R	CCGACAATACAAGCACCTATTCTCC
arcR-F	CTTGGATTGCCTAGAGAATTGATGG
arcR-R	AGTTCATGTGTTGCTGCTCATTATC
yghA-F	ACAGGTGGTGACTCAGCAATAGG
yghA-R	TGGCGTACTTCTTGTGCATCTTG

3. Supplementary Notes

Supplementary Note 1. Synthetic scheme for broad-spectrum antibacterial small molecule compounds based on SBDD and modular synthesis

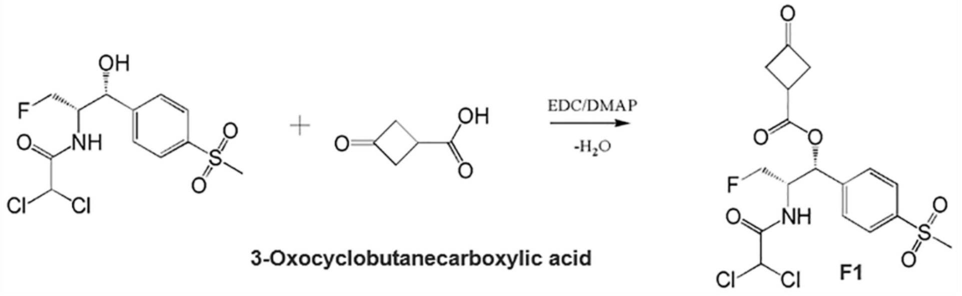

Scheme S1. Synthetic scheme for F1.

Reagents and conditions: florfenicol, 3-oxocyclobutanecarboxylic acid, EDC, DMAP, 25℃, 24 h, 46%.

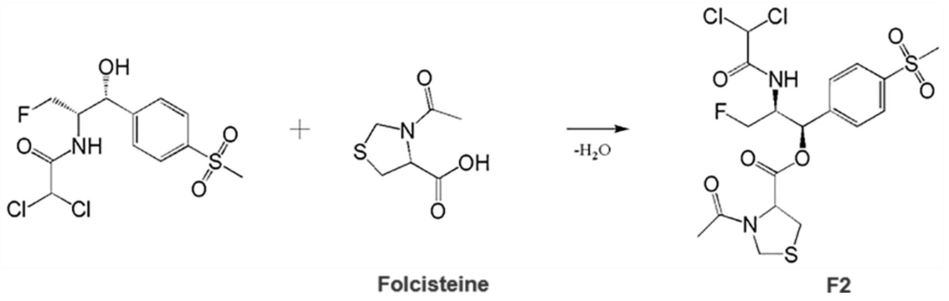

Scheme S2. Synthetic scheme for F2.

Reagents and conditions: florfenicol, folcisteine, EDC, DMAP, 25℃, 24 h, 62%.

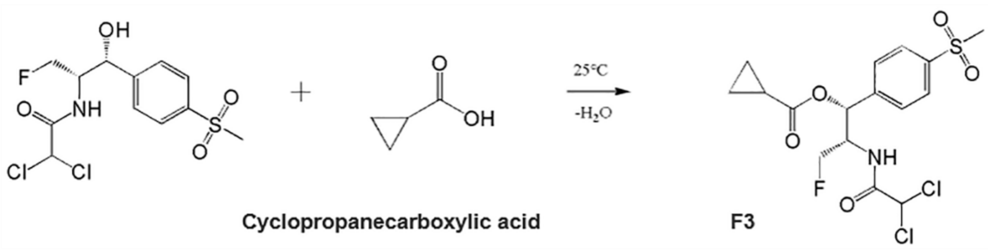

Scheme S3. Synthetic scheme for F3.

Reagents and conditions: florfenicol, cyclopropanecarboxylic acid, EDC, DMAP, 25℃, 24 h, 51%.

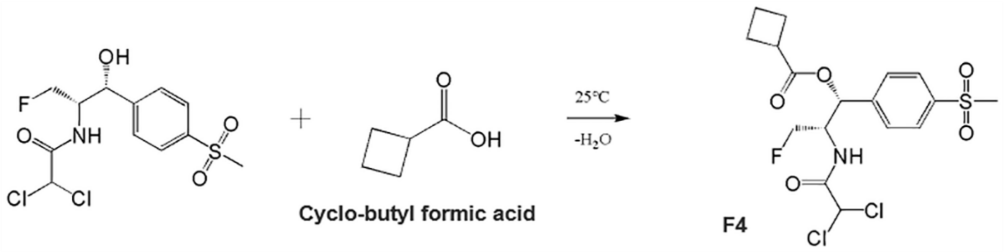

Scheme S4. Synthetic scheme for F4.

Reagents and conditions: florfenicol, cyclo-butyl formic acid, EDC, DMAP, 25°C, 24 h, 42%.

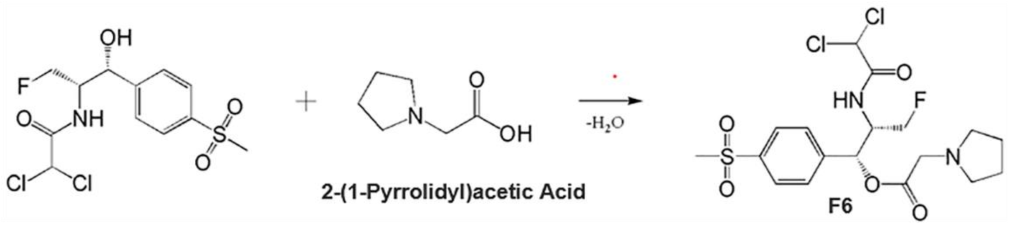

Scheme S5. Synthetic scheme for F6.

Reagents and conditions: florfenicol, 2-(1-pyrrolidyl)acetic acid, EDC, DMAP, 25°C, 24 h, 39%.

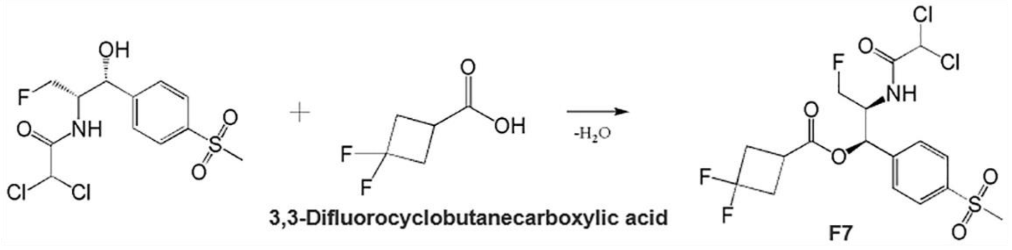

Scheme S6. Synthetic scheme for F7.

Reagents and conditions: florfenicol, 3,3-difluorocyclobutanecarboxylic acid, EDC, DMAP, 25°C, 24 h, 39%.

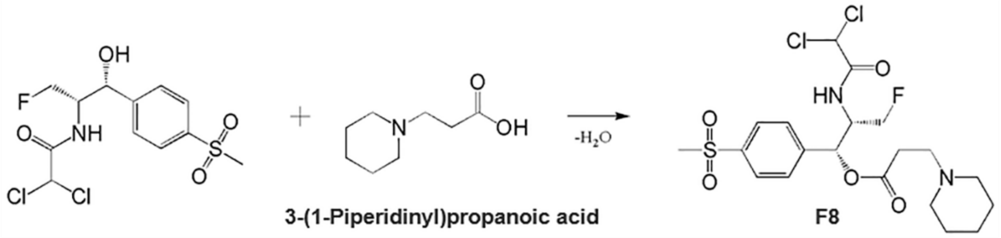

Scheme S7. Synthetic scheme for F8.

Reagents and conditions: florfenicol, 3-(1-piperidinyl)propanoic acid, EDC, DMAP, 25℃, 24 h, 41%.

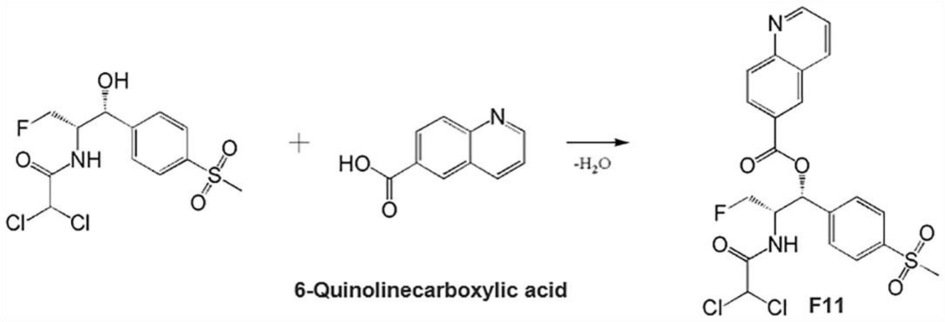

Scheme S8. Synthetic scheme for F11.

Reagents and conditions: florfenicol, 6-quinolinecarboxylic acid, EDC, DMAP, 25℃, 24 h, 45%.

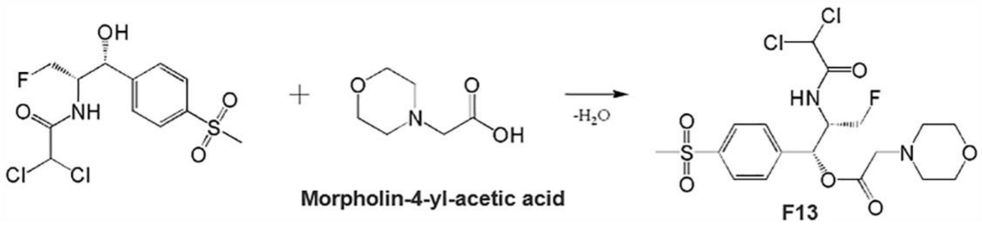

Scheme S9. Synthetic scheme for F13.

Reagents and conditions: florfenicol, morpholin-4-yl-acetic acid, EDC, DMAP, 25℃, 24 h, 41%.

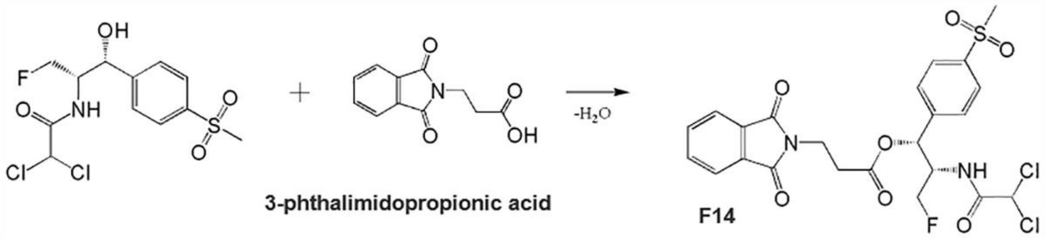

Scheme S10. Synthetic scheme for F14.

Reagents and conditions: florfenicol, 3-phthalimidopropionic acid, EDC, DMAP, 25℃, 24 h, 52%.

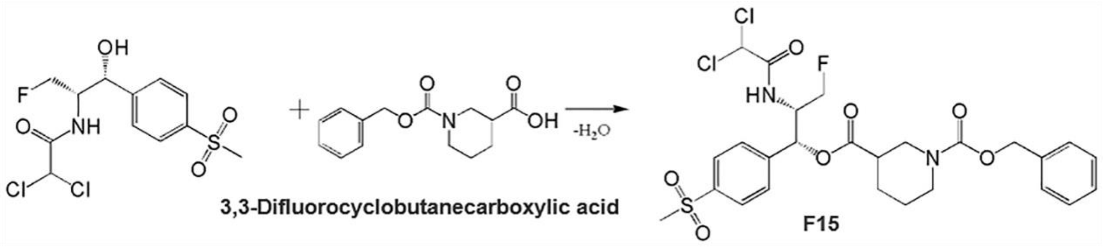

Scheme S11. Synthetic scheme for F15.

Reagents and conditions: florfenicol, 3,3-difluorocyclobutanecarboxylic acid, EDC, DMAP, 25℃, 24 h, 42%.

Supplementary Note 2. MS data of the final compounds

High-resolution mass analysis was performed on the liquid chromatograph mass spectrometry–ion trap–time of flight (LCMS–IT–TOF) from Shimadzu (Kyoto, Japan).

F1, m/z calculated for $C_{17}H_{18}Cl_2FNO_6S$ $[M+H]^+$: 453.0216, found: 452.0009

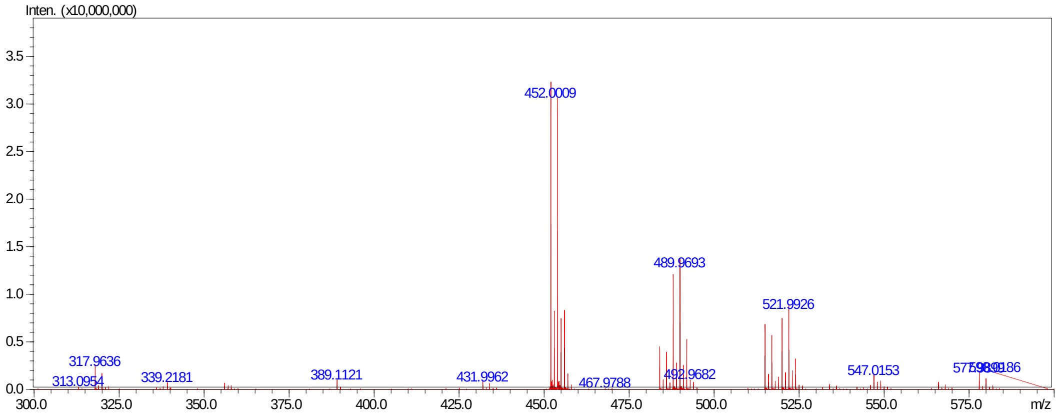

F2, m/z calculated for $C_{18}H_{21}Cl_2FN_2O_6S_2$ $[M+H]^+$: 514.0202, found: 514.9950

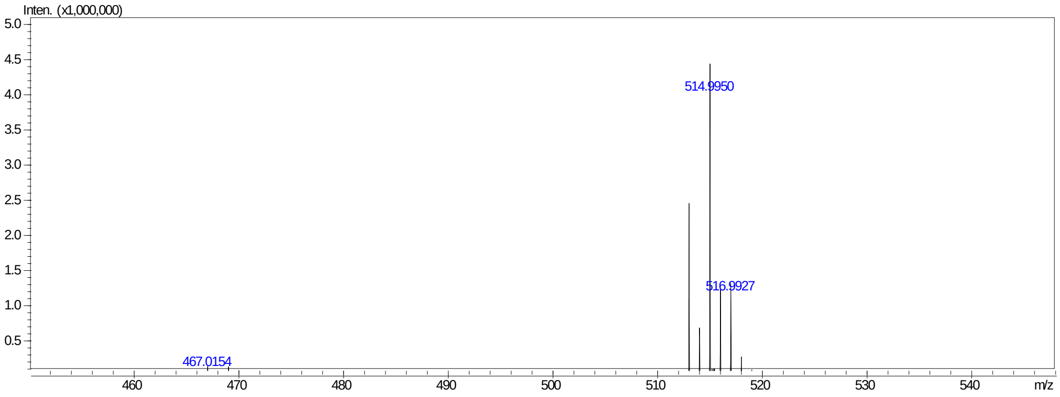

F3, m/z calculated for $C_{16}H_{18}Cl_2FNO_5S$ $[M+H]^+$: 425.0267, found: 424.0068

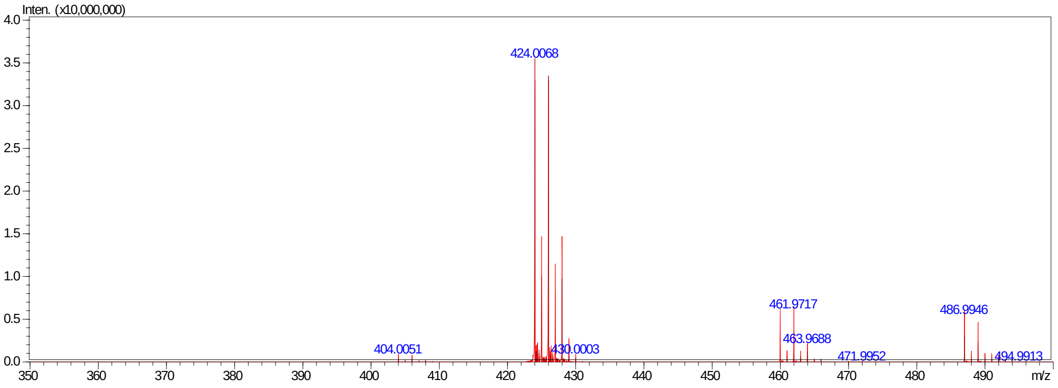

F4, m/z calculated for $C_{17}H_{20}Cl_2FNO_5S$ $[M+H]^+$: 440.3138, found: 440.0529

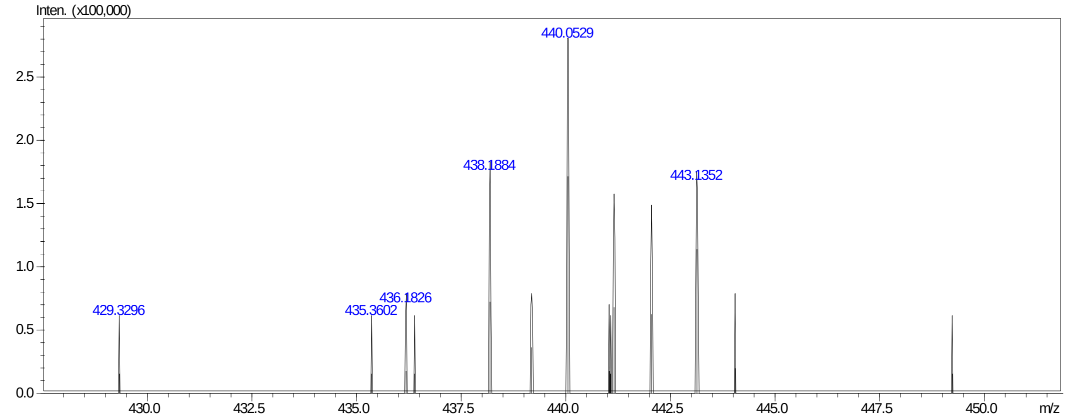

F6, m/z calculated for $C_{18}H_{23}Cl_2FN_2O_5S$ $[M+H]^+$: 468.0689, found: 467.0515

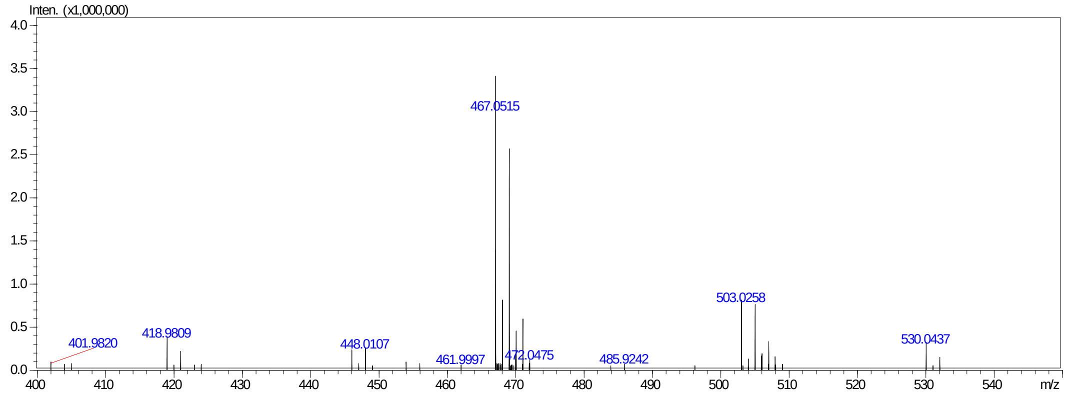

F7, m/z calculated for $C_{17}H_{18}Cl_2F_3N_1O_5S$ $[M+H]^+$: 475.0235, found: 475.3320

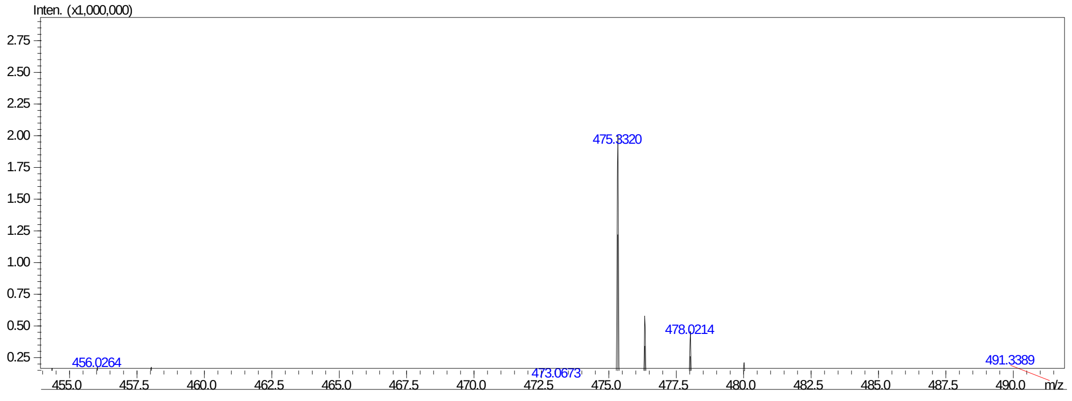

F8, m/z calculated for $C_{20}H_{27}Cl_2FN_2O_5S$ $[M+H]^+$: 497.4082, found: 497.0945

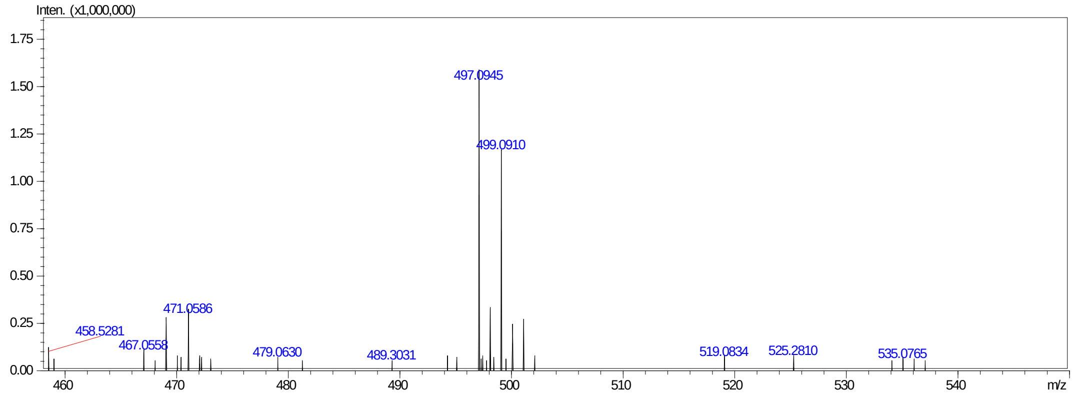

F11, m/z calculated for $C_{22}H_{19}Cl_2FN_2O_5S$ $[M+H]^+$: 513.3661, found: 513.0349

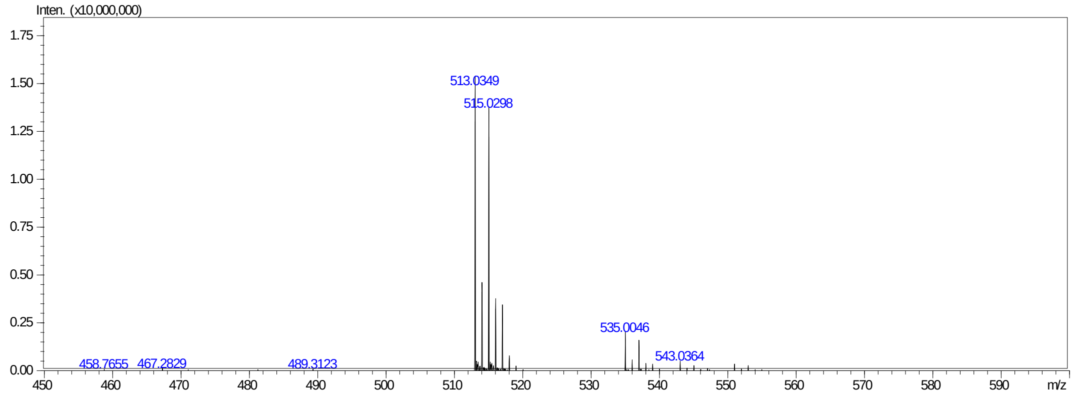

F13, m/z calculated for $C_{18}H_{23}Cl_2FN_2O_6S$ $[M+H]^+$: 485.3544, found: 485.0699

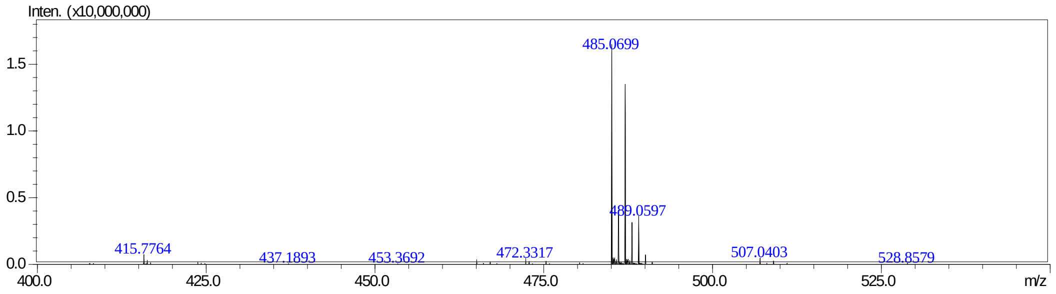

F14, m/z calculated for $C_{23}H_{21}Cl_2FN_2O_7S$ $[M+H]^+$: 559.3914, found: 559.0531

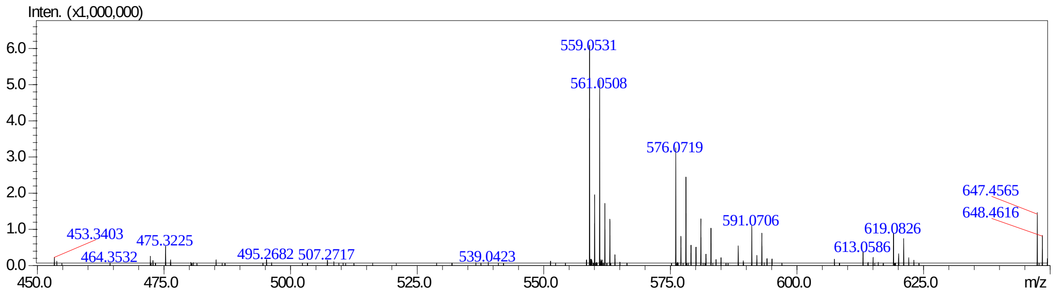

F15, m/z calculated for $C_{26}H_{29}Cl_2FN_2O_7S$ $[M+H]^+$: 603.4871, found: 6603.1110

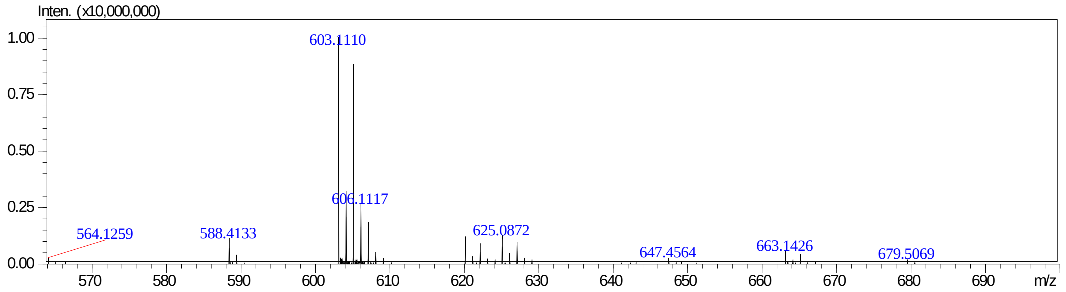

Supplementary Note 3. NMR spectra of synthetic compounds

The ^{1}H- and ^{13}C-NMR spectra were recorded on a Bruker Avance 400 spectrometer at 25°C using DMSO-α₆ as the solvent. Chemical shifts (δ) are reported in ppm relative to Me_4Si (internal standard), coupling constants (*J*) are reported in hertz, and peak multiplicity is reported as s (singlet), d (doublet), t (triplet), q (quartet), m (multiplet), or br s (broad singlet).

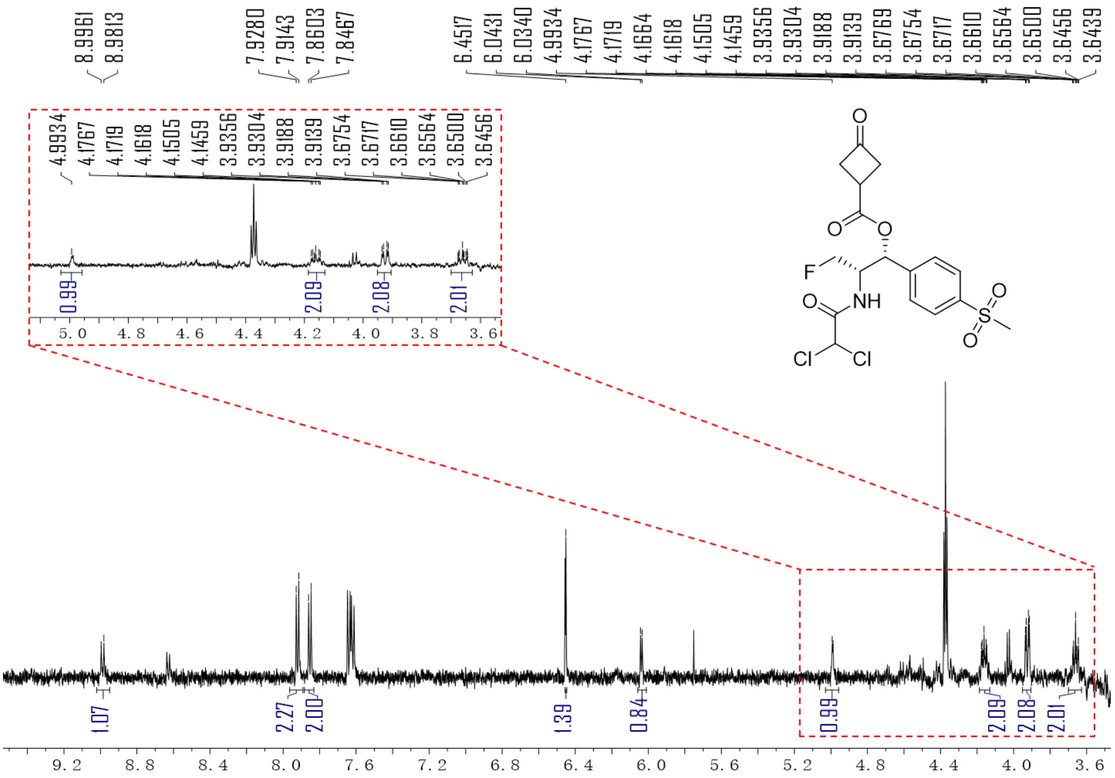

^{1}H-NMR spectrum of compound F1 (600 MHz, DMSO).

^{1}H NMR (600 MHz, DMSO-d_6) δ 8.99 (m, 1H), 7.92 (d, *J* = 8.3 Hz, 2H), 7.85 (d, *J* = 8.2 Hz, 2H), 6.45 (s, 1H), 6.04 (m, 1H), 4.99 (m, 1H), 4.19 – 4.13 (m, 2H), 3.92 (m, 2H), 3.70 – 3.63 (m, 2H).

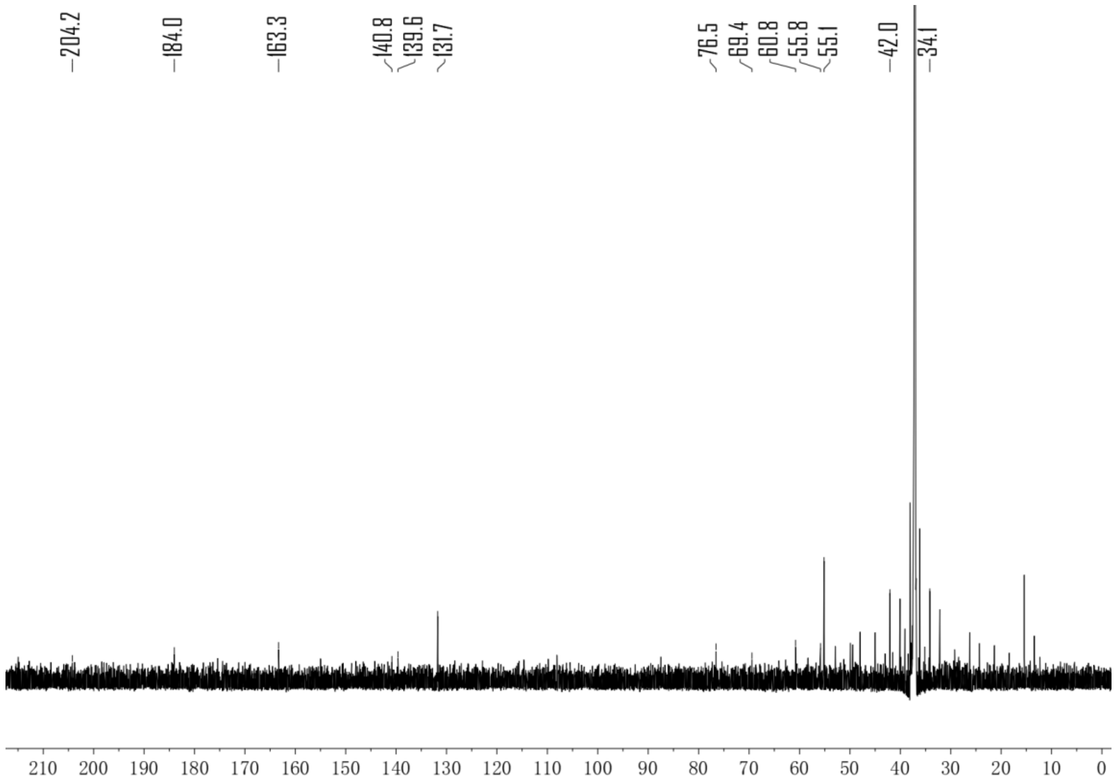

^{13}C-NMR spectrum of compound F1 (150 MHz, DMSO).

^{13}C NMR (150 MHz, DMSO) δ 204.22, 184.01, 163.31, 140.84, 139.61, 131.75, 76.55, 69.42, 60.77, 55.83, 55.13, 42.03, 34.15.

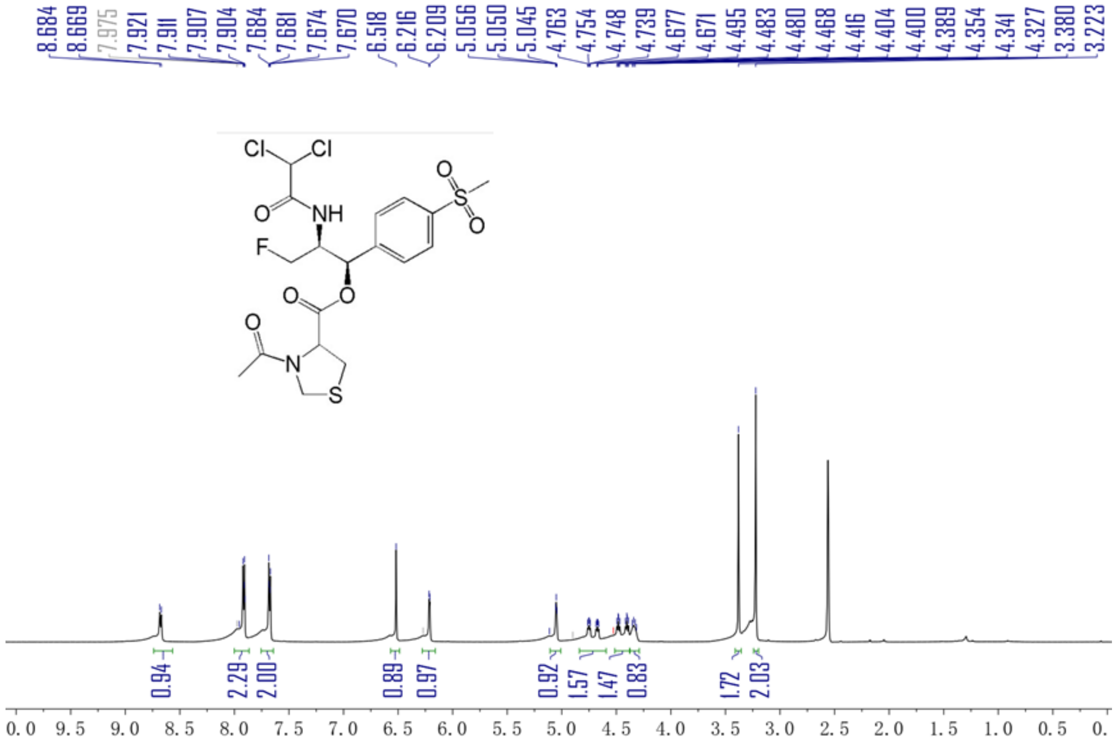

^{1}H-NMR spectrum of compound F2 (600 MHz, DMSO).

^{1}H NMR (600 MHz, DMSO-d_6) δ 8.68 (d, J = 9.0 Hz, 1H), 8.00 – 7.87 (m, 2H), 7.76 – 7.64 (m, 2H), 6.52 (s, 1H), 6.21 (d, J = 4.2 Hz, 1H), 5.05 (t, J = 3.5 Hz, 1H), 4.71 (ddd, J = 46.3, 9.2, 5.5 Hz, 2H), 4.44 (ddd, J = 47.5, 9.3, 7.2 Hz, 1H), 4.33 (q, J = 7.4, 7.0 Hz, 1H), 3.38 (s, 2H), 3.22 (s, 2H).

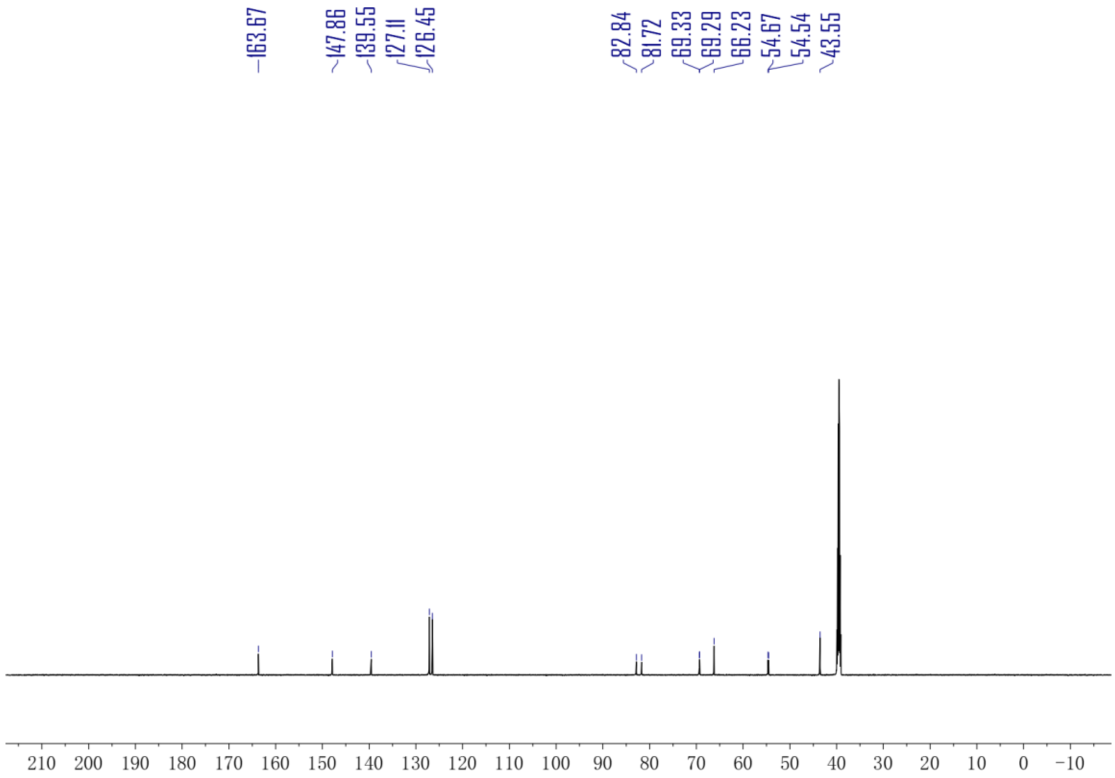

^{13}C-NMR spectrum of compound F2 (150 MHz, DMSO).

^{13}C NMR (150 MHz, DMSO) δ 163.67, 147.86, 139.55, 127.11, 126.45, 82.84, 81.72, 69.33, 69.29, 66.23, 54.67, 54.54, 43.55.

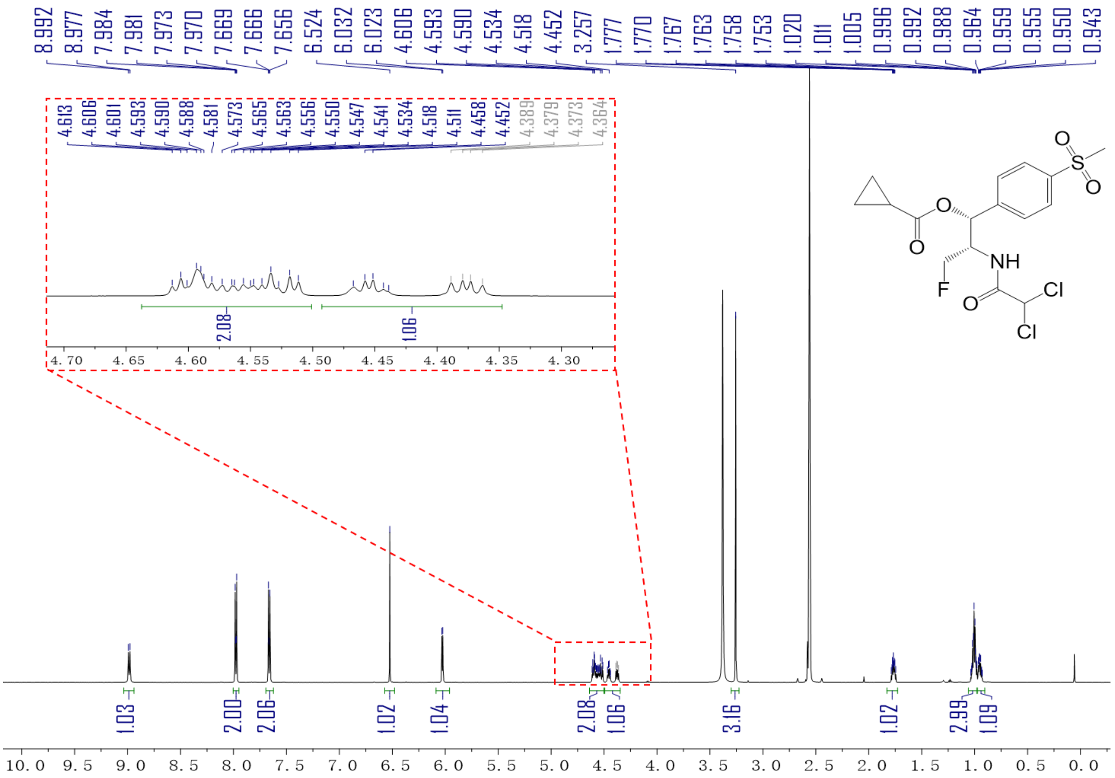

^{1}H-NMR spectrum of compound F3 (600 MHz, DMSO).

^{1}H NMR (600 MHz, DMSO-d_6) δ 8.98 (d, *J* = 8.8 Hz, 1H), 8.00 – 7.95 (m, 2H), 7.66 (d, *J* = 8.2 Hz, 2H), 6.52 (s, 1H), 6.03 (d, *J* = 5.3 Hz, 1H), 4.64 – 4.50 (m, 2H), 4.45 (dt, *J* = 7.6, 4.1 Hz, 1H), 3.26 (s, 3H), 1.77 (tt, *J* = 7.8, 4.7 Hz, 1H), 1.00 (td, *J* = 9.7, 9.2, 6.1 Hz, 3H), 0.97 – 0.90 (m, 1H).

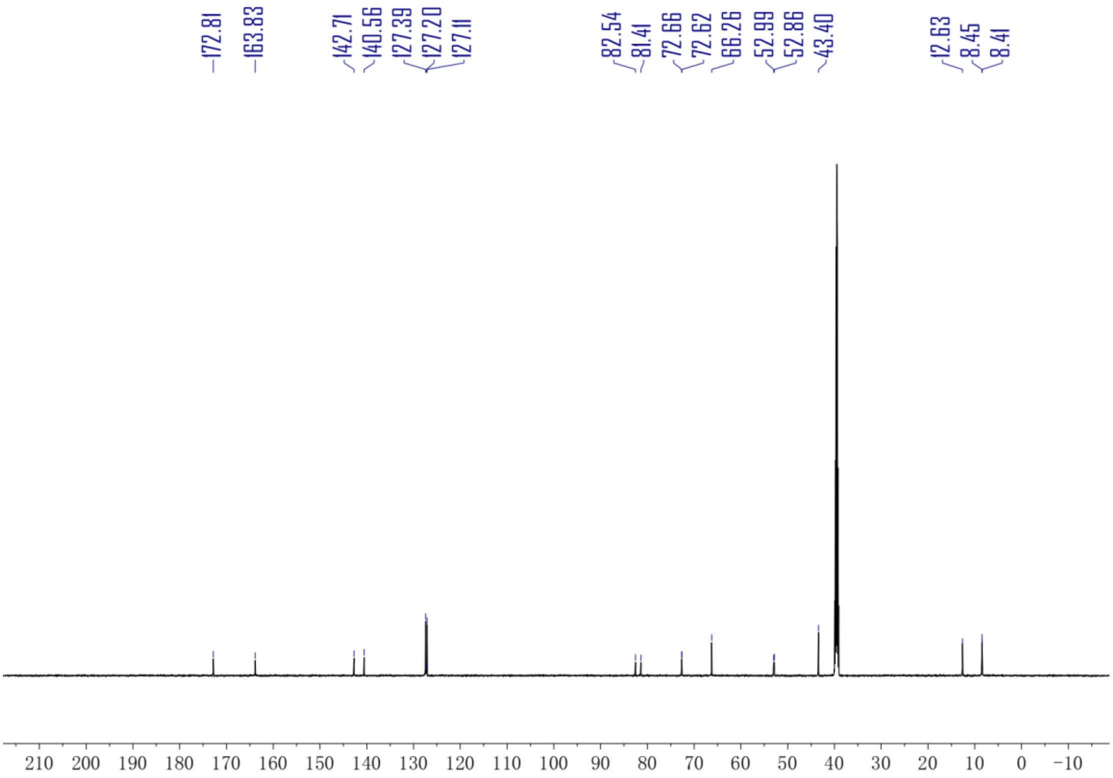

^{13}C-NMR spectrum of compound F3 (150 MHz, DMSO).

^{13}C NMR (150 MHz, DMSO) δ 172.81, 163.83, 142.71, 140.56, 127.39, 127.20, 127.11, 82.54, 81.41, 72.66, 72.62, 66.26, 52.99, 52.86, 43.40, 12.63, 8.45, 8.41.

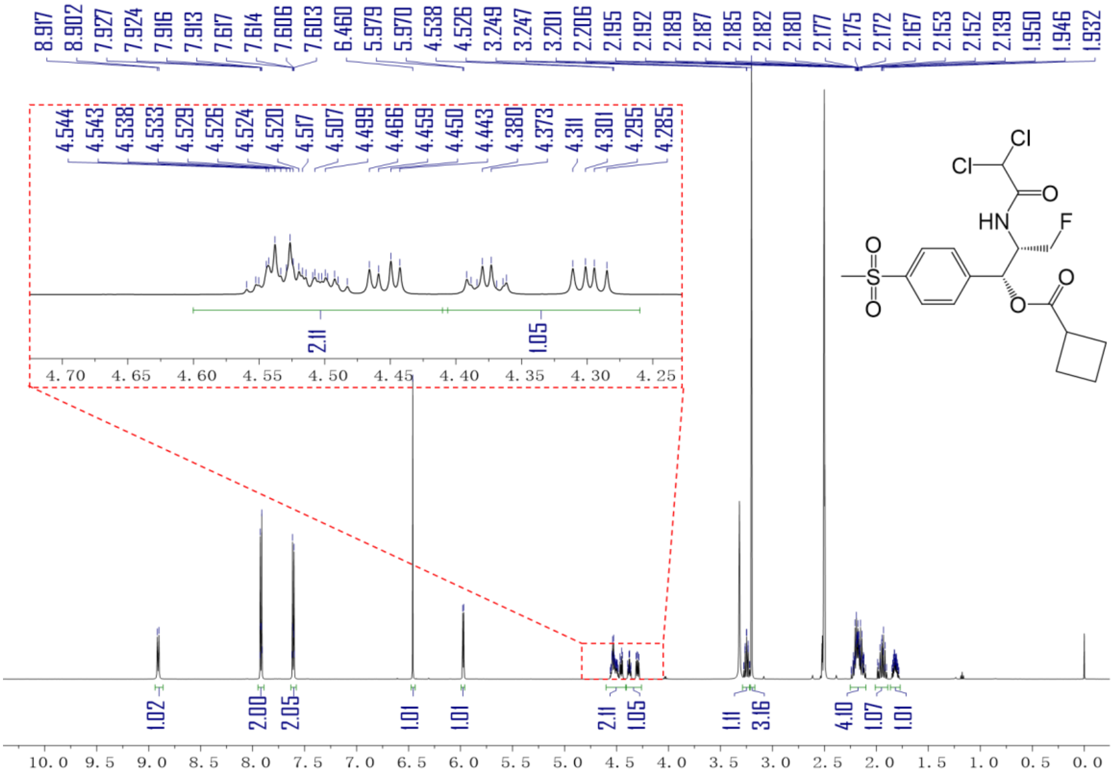

^{1}H-NMR spectrum of compound F4 (600 MHz, DMSO).

^{1}H NMR (600 MHz, DMSO-d_6) δ 8.91 (d, *J* = 8.8 Hz, 1H), 7.95 – 7.89 (m, 2H), 7.63 – 7.58 (m, 2H), 6.46 (s, 1H), 5.97 (d, *J* = 5.7 Hz, 1H), 4.60 – 4.41 (m, 2H), 4.41 – 4.26 (m, 1H), 3.29 – 3.21 (m, 1H), 3.20 (s, 3H), 2.25 – 2.10 (m, 4H), 2.01 – 1.89 (m, 1H), 1.82 (ddddd, *J* = 10.1, 9.0, 6.5, 4.5, 1.1 Hz, 1H).

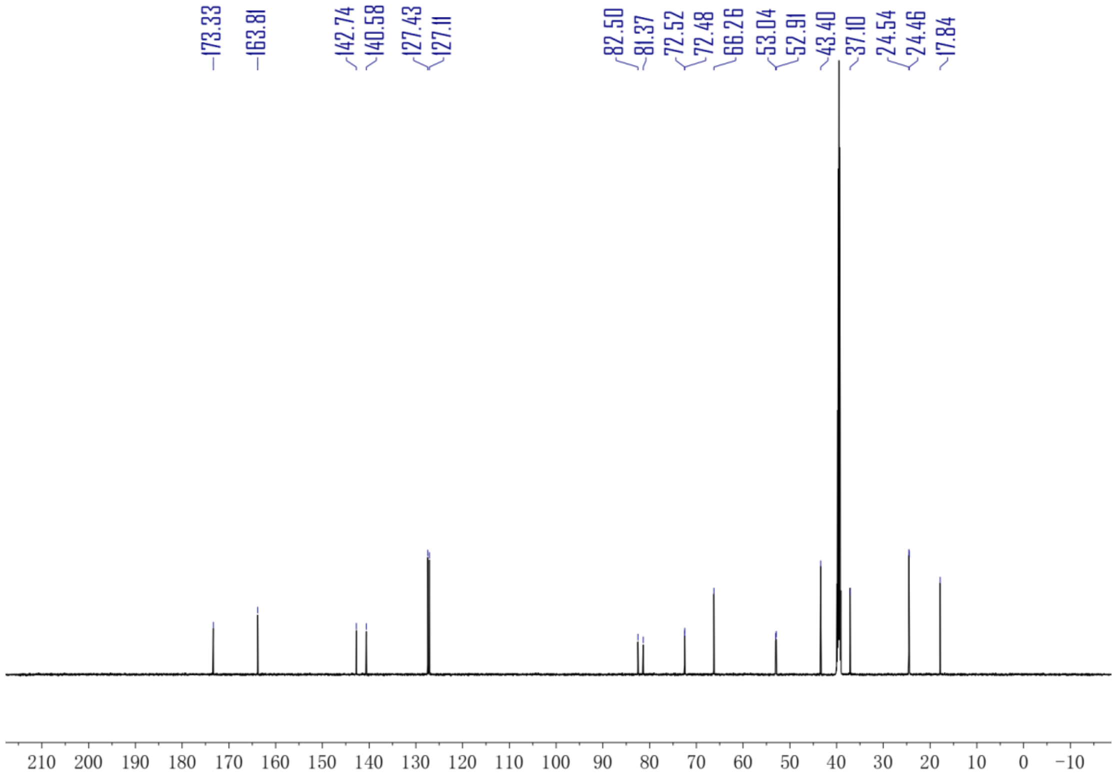

^{13}C-NMR spectrum of compound F4 (150 MHz, DMSO).

^{13}C NMR (150 MHz, DMSO) δ 173.33, 163.81, 142.74, 140.58, 127.43, 127.11, 82.50, 81.37, 72.52, 72.48, 66.26, 53.04, 52.91, 43.40, 37.10, 24.54, 24.46, 17.84.

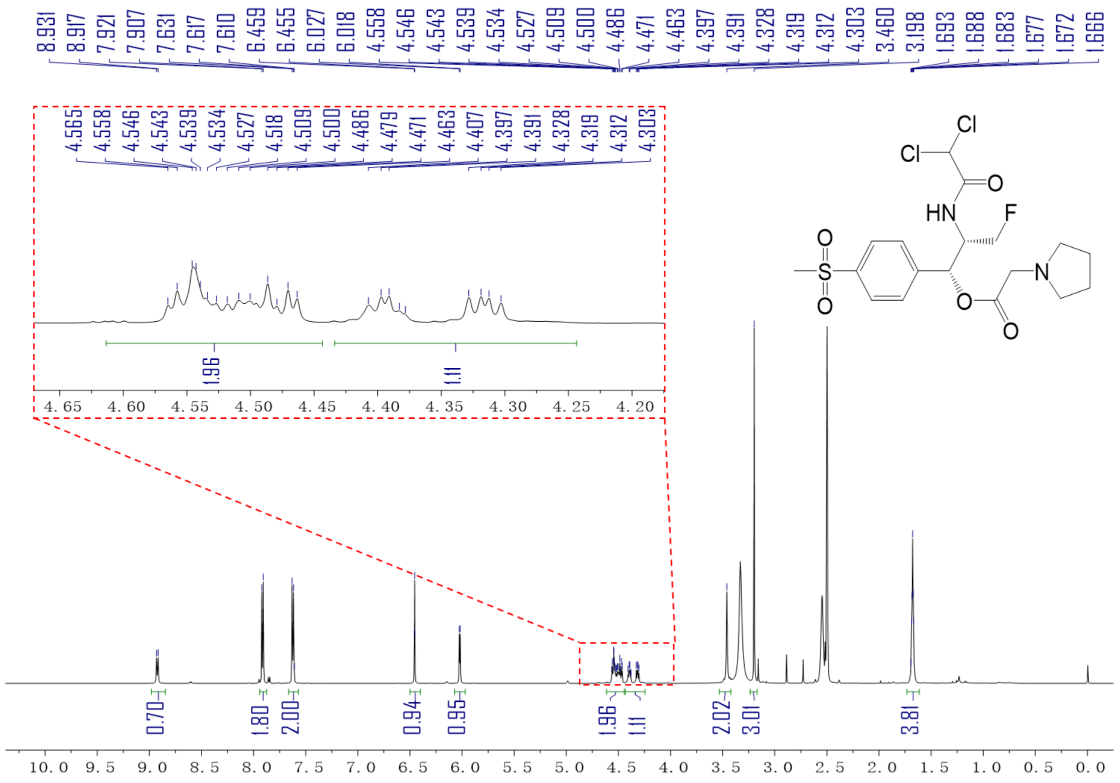

^{1}H-NMR spectrum of compound F6 (600 MHz, DMSO).

^{1}H NMR (600 MHz, DMSO-d_6) δ 8.92 (d, *J* = 8.8 Hz, 1H), 7.91 (d, *J* = 8.2 Hz, 2H), 7.62 (d, *J* = 8.2 Hz, 2H), 6.46 (d, *J* = 2.4 Hz, 1H), 6.02 (d, *J* = 5.1 Hz, 1H), 4.61 – 4.44 (m, 2H), 4.36 (ddd, *J* = 48.0, 9.6, 5.8 Hz, 1H), 3.46 (s, 2H), 3.20 (s, 3H), 2.55 (s, 4H), 1.68 (h, *J* = 3.2 Hz, 4H).

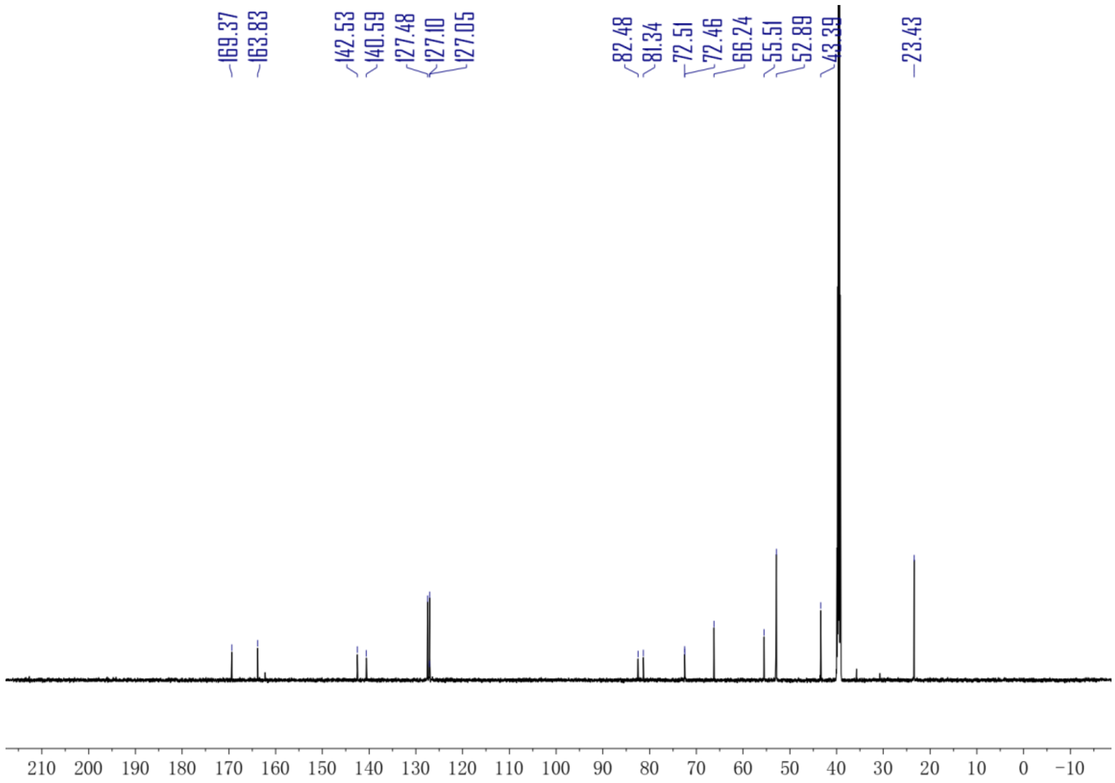

^{13}C-NMR spectrum of compound F6 (150 MHz, DMSO).

^{13}C NMR (150 MHz, DMSO) δ 169.37, 163.83, 142.53, 140.59, 127.48, 127.10, 127.05, 82.48, 81.34, 72.51, 72.46, 66.24, 55.51, 52.89, 43.39, 23.43.

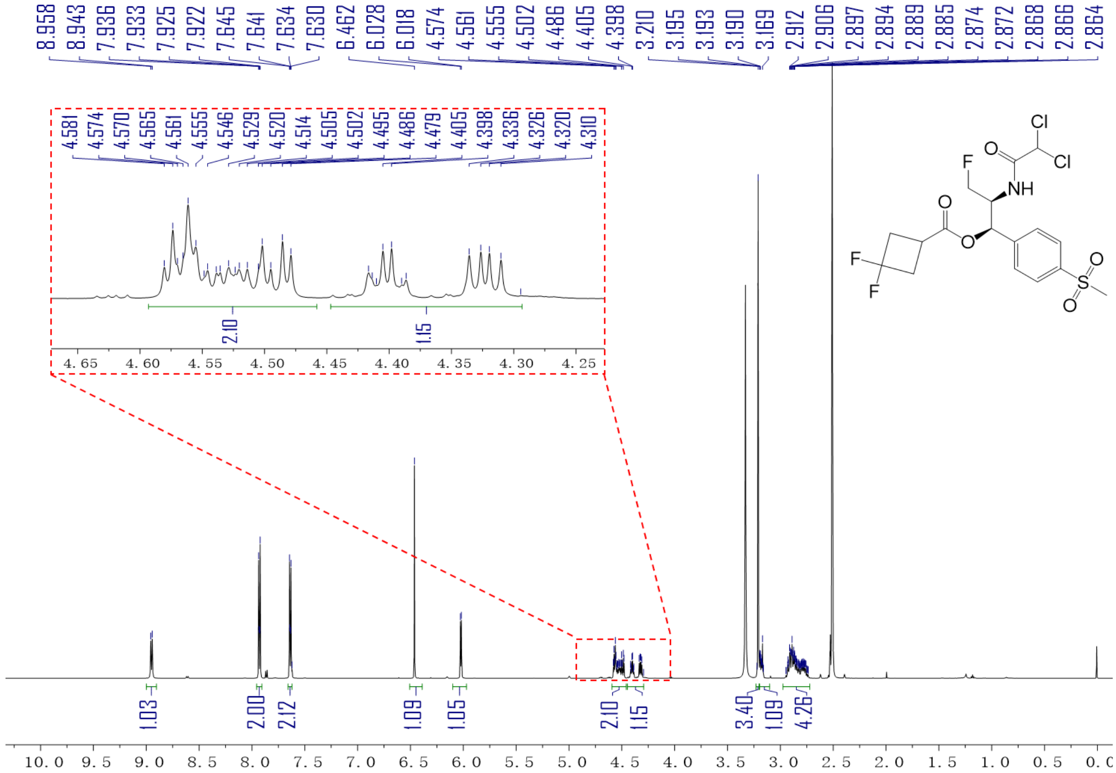

^{1}H-NMR spectrum of compound F7 (600 MHz, DMSO).

^{1}H NMR (600 MHz, DMSO-d_6) δ 8.95 (d, *J* = 8.7 Hz, 1H), 7.96 – 7.91 (m, 2H), 7.66 – 7.62 (m, 2H), 6.46 (s, 1H), 6.02 (d, *J* = 5.6 Hz, 1H), 4.59 – 4.46 (m, 2H), 4.45 – 4.29 (m, 1H), 3.21 (s, 3H), 3.20 – 3.10 (m, 1H), 2.98 – 2.72 (m, 4H).

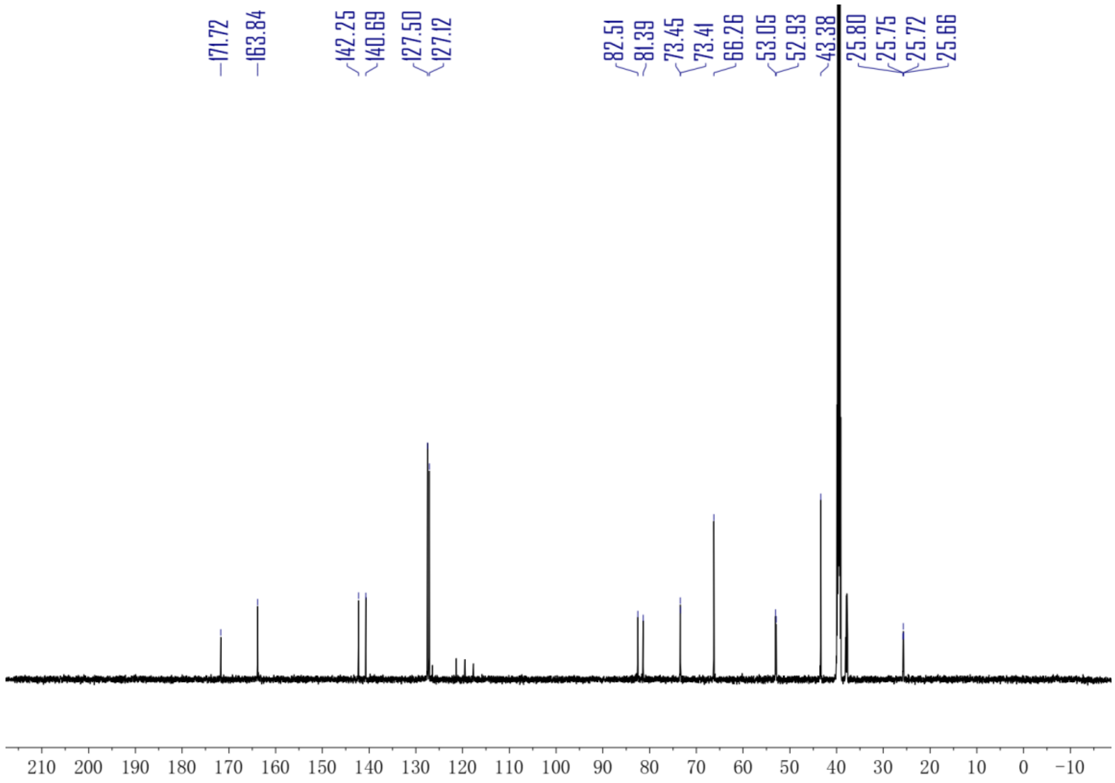

^{13}C-NMR spectrum of compound F7 (150 MHz, DMSO).

^{13}C NMR (150 MHz, DMSO) δ 171.72, 163.84, 142.25, 140.69, 127.50, 127.12, 82.51, 81.39, 73.45, 73.41, 66.26, 53.05, 52.93, 43.38, 25.80, 25.75, 25.72, 25.66.

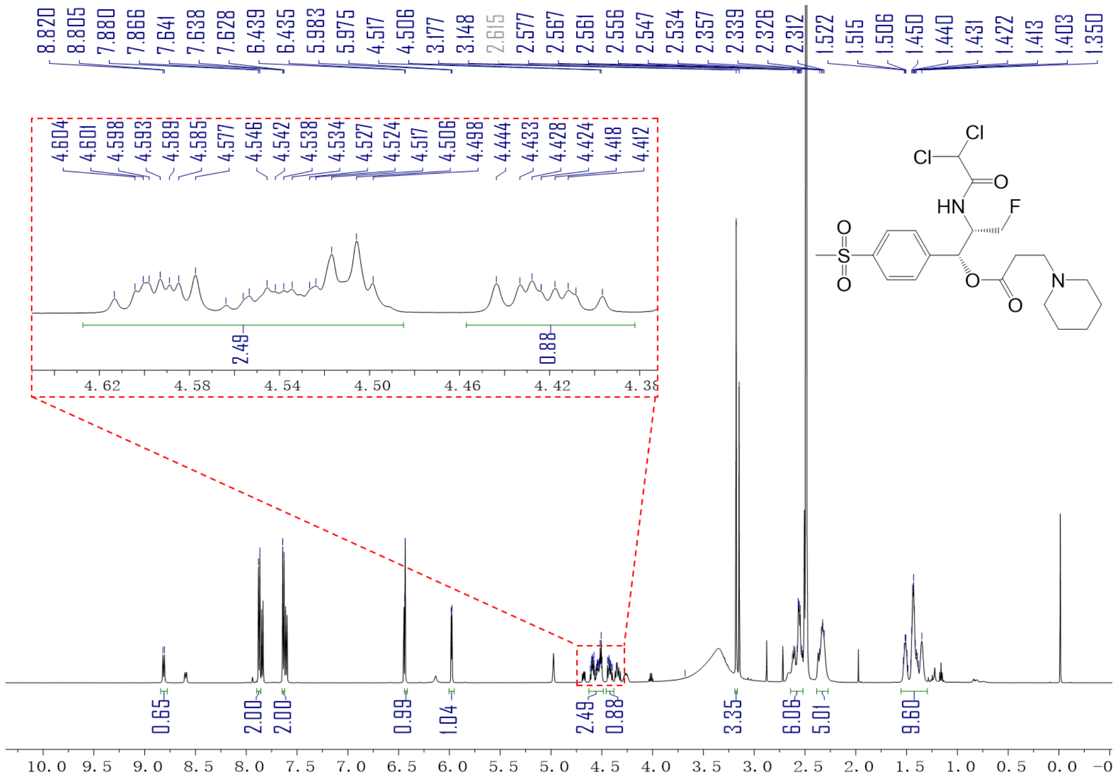

^{1}H-NMR spectrum of compound F8 (600 MHz, DMSO).

^{1}H NMR (600 MHz, DMSO-d_6) δ 8.81 (d, *J* = 8.7 Hz, 1H), 7.87 (d, *J* = 8.1 Hz, 2H), 7.63 (d, *J* = 8.3 Hz, 2H), 6.44 (d, *J* = 2.4 Hz, 1H), 5.98 (d, *J* = 4.5 Hz, 1H), 4.63 – 4.48 (m, 2H), 4.42 (ddd, *J* = 12.6, 9.3, 6.7 Hz, 1H), 3.18 (s, 3H), 2.64 – 2.52 (m, 6H), 2.33 (dd, *J* = 17.4, 9.1 Hz, 5H), 1.55 – 1.30 (m, 6H).

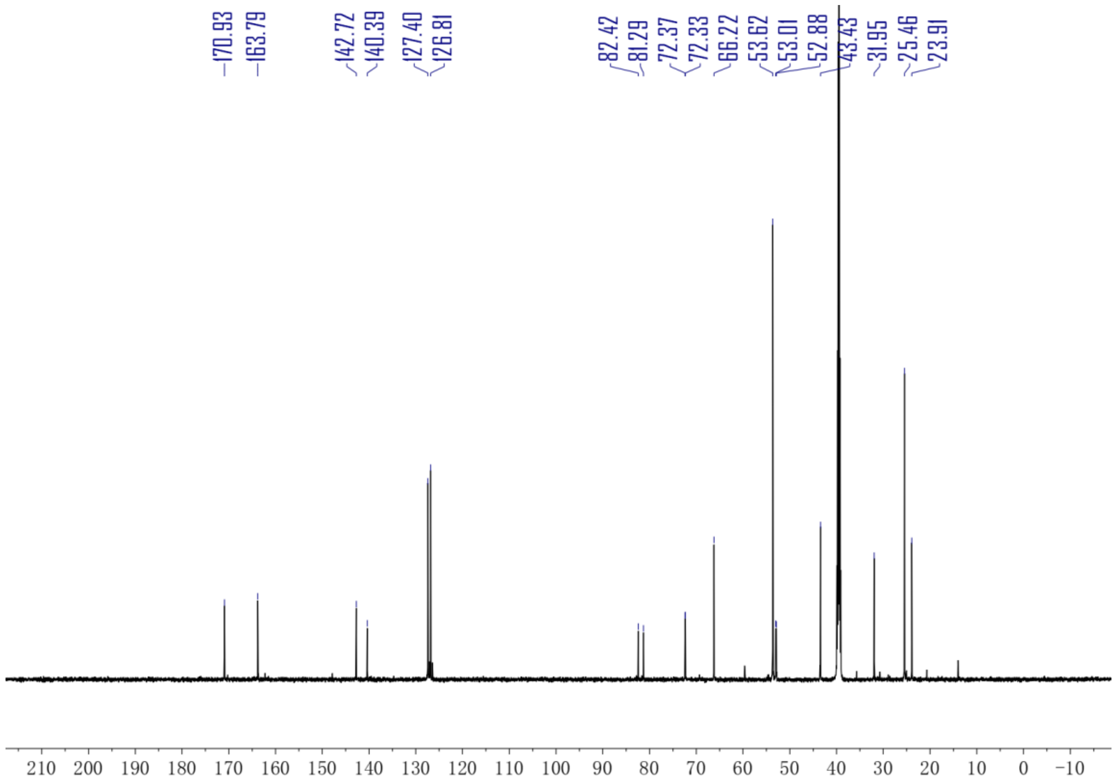

^{13}C-NMR spectrum of compound F8 (150 MHz, DMSO).

^{13}C NMR (150 MHz, DMSO) δ 170.93, 163.79, 142.72, 140.39, 127.40, 126.81, 82.42, 81.29, 72.37, 72.33, 66.22, 53.62, 53.01, 52.88, 43.43, 31.95, 25.46, 23.91.

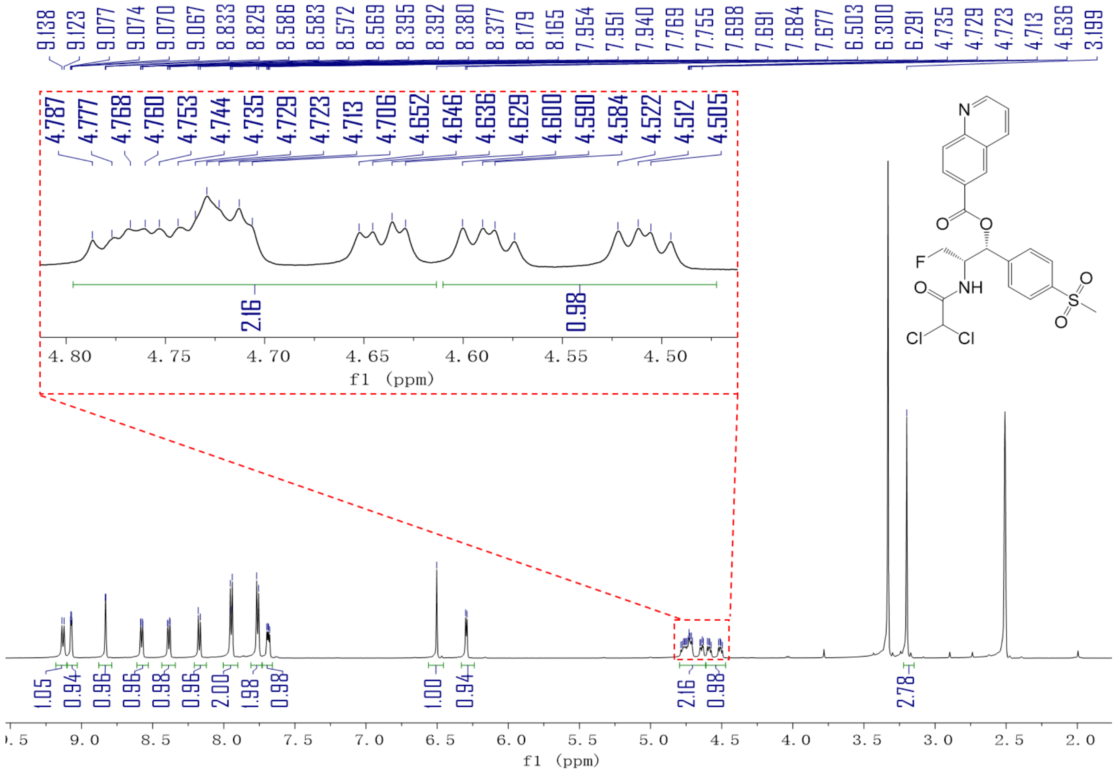

^{1}H-NMR spectrum of compound F11 (600 MHz, DMSO).

^{1}H NMR (600 MHz, DMSO-d_6) δ 9.13 (d, *J* = 9.1 Hz, 1H), 9.07 (dd, *J* = 4.3, 1.9 Hz, 1H), 8.83 (d, *J* = 2.0 Hz, 1H), 8.58 (dd, *J* = 8.4, 1.7 Hz, 1H), 8.39 (dd, *J* = 8.8, 2.0 Hz, 1H), 8.17 (d, *J* = 8.8 Hz, 1H), 7.95 (d, *J* = 8.2 Hz, 2H), 7.76 (d, *J* = 8.2 Hz, 2H), 7.69 (dd, *J* = 8.3, 4.2 Hz, 1H), 6.50 (s, 1H), 6.30 (d, *J* = 5.6 Hz, 1H), 4.80 – 4.61 (m, 2H), 4.55 (ddd, *J* = 47.3, 9.9, 6.1 Hz, 1H), 3.20 (s, 3H).

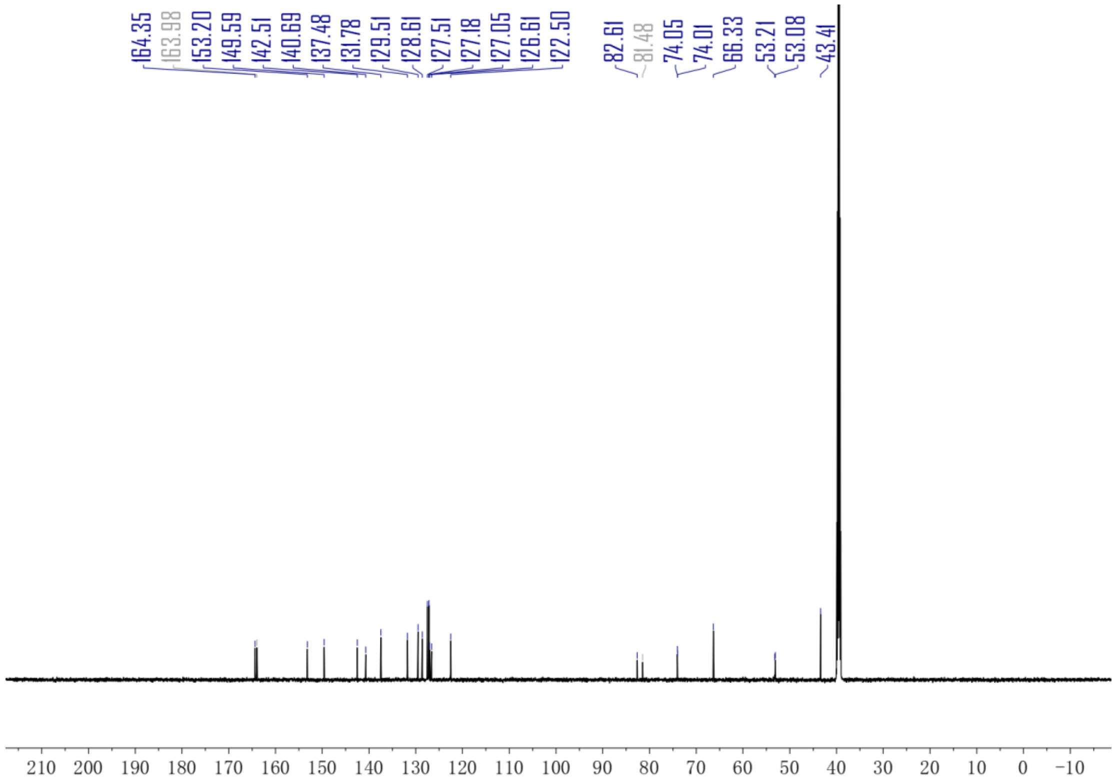

^{13}C-NMR spectrum of compound F11 (150 MHz, DMSO).

^{13}C NMR (150 MHz, DMSO) δ 164.35, 163.98, 153.20, 149.59, 142.51, 140.69, 137.48, 131.78, 129.51, 128.61, 127.51, 127.18, 127.05, 126.61, 122.50, 82.61, 81.48, 74.05, 74.01, 66.33, 53.21, 53.08, 43.41.

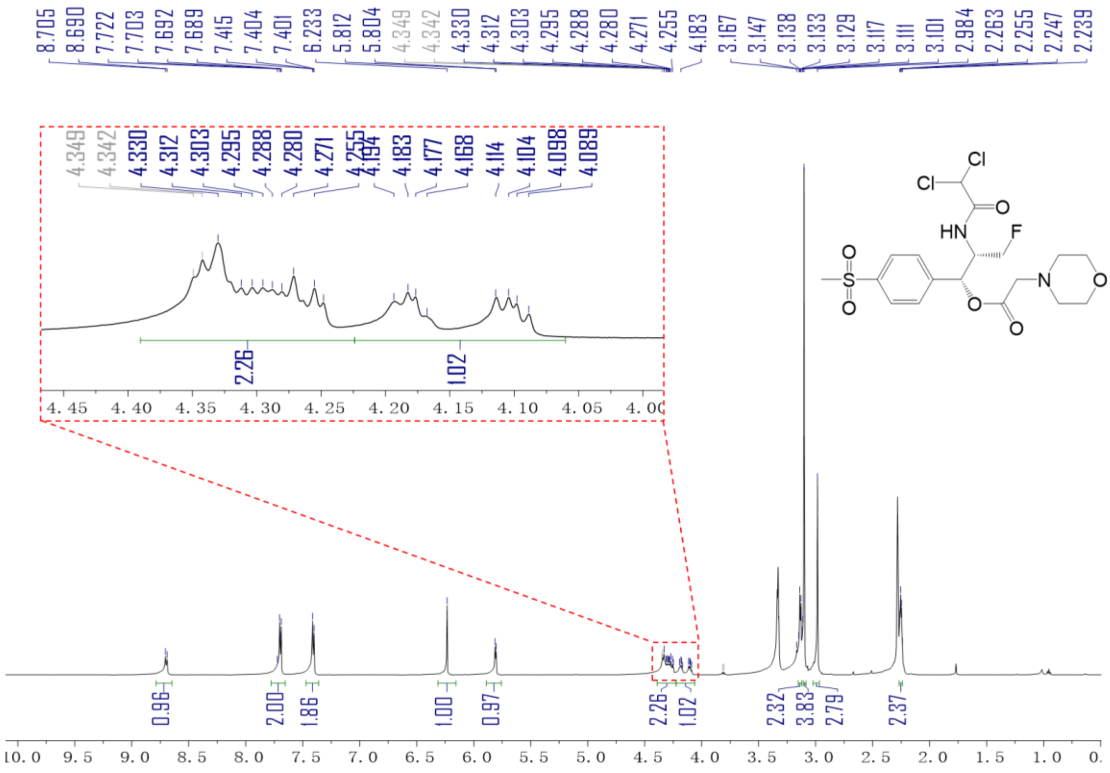

^{1}H-NMR spectrum of compound F13 (600 MHz, DMSO).

^{1}H NMR (600 MHz, DMSO-d_6) δ 8.70 (d, *J* = 8.9 Hz, 1H), 7.70 (dd, *J* = 13.2, 6.6 Hz, 2H), 7.41 (d, *J* = 8.5 Hz, 2H), 6.23 (s, 1H), 5.81 (d, *J* = 5.2 Hz, 1H), 4.39 – 4.22 (m, 2H), 4.14 (ddd, *J* = 47.4, 9.8, 6.1 Hz, 1H), 3.15 – 3.12 (m, 2H), 3.10 (s, 4H), 2.98 (s, 3H), 2.27 – 2.24 (m, 2H).

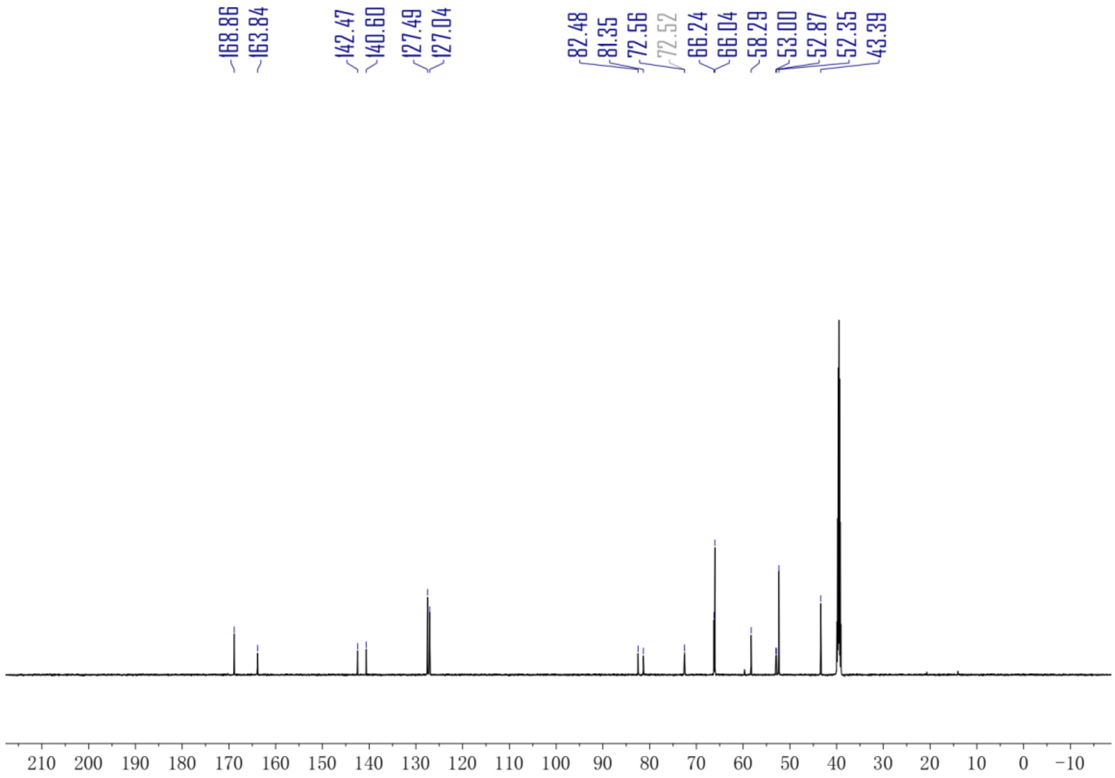

^{13}C-NMR spectrum of compound F13 (150 MHz, DMSO).

^{13}C NMR (150 MHz, DMSO) δ 168.86, 163.84, 142.47, 140.60, 127.49, 127.04, 82.48, 81.35, 72.56, 72.52, 66.24, 66.04, 58.29, 53.00, 52.87, 52.35, 43.39.

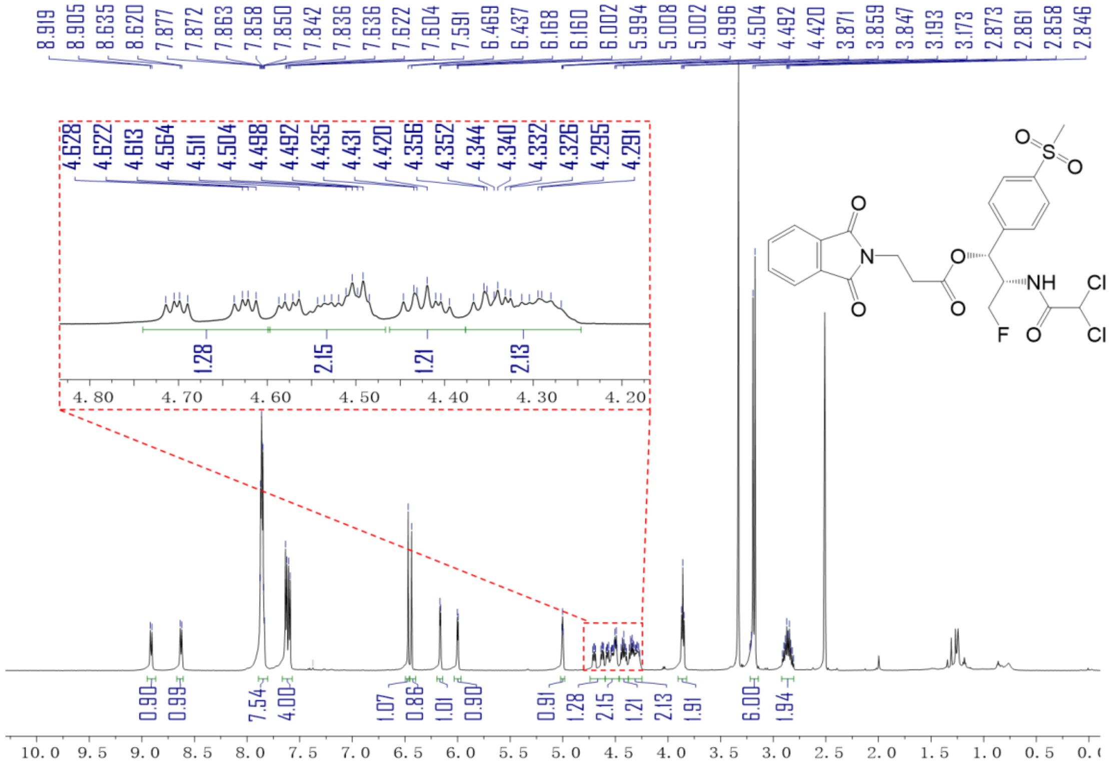

^{1}H-NMR spectrum of compound F14 (600 MHz, DMSO).

^{1}H NMR (600 MHz, DMSO-d_6) δ 8.91 (d, *J* = 8.7 Hz, 1H), 8.63 (d, *J* = 9.0 Hz, 1H), 7.86 (dt, *J* = 9.5, 5.0 Hz, 8H), 7.61 (dd, *J* = 19.0, 8.1 Hz, 4H), 6.47 (s, 1H), 6.44 (s, 1H), 6.16 (d, *J* = 4.2 Hz, 1H), 6.00 (d, *J* = 5.0 Hz, 1H), 5.00 (t, *J* = 3.6 Hz, 1H), 4.66 (ddd, *J* = 46.3, 9.2, 5.5 Hz, 1H), 4.60 – 4.47 (m, 2H), 4.42 (ddd, *J* = 15.2, 9.3, 6.5 Hz, 1H), 4.38 – 4.25 (m, 2H), 3.86 (t, *J* = 7.1 Hz, 2H), 3.18 (d, *J* = 11.8 Hz, 6H), 2.86 (qt, *J* = 16.5, 7.2 Hz, 2H).

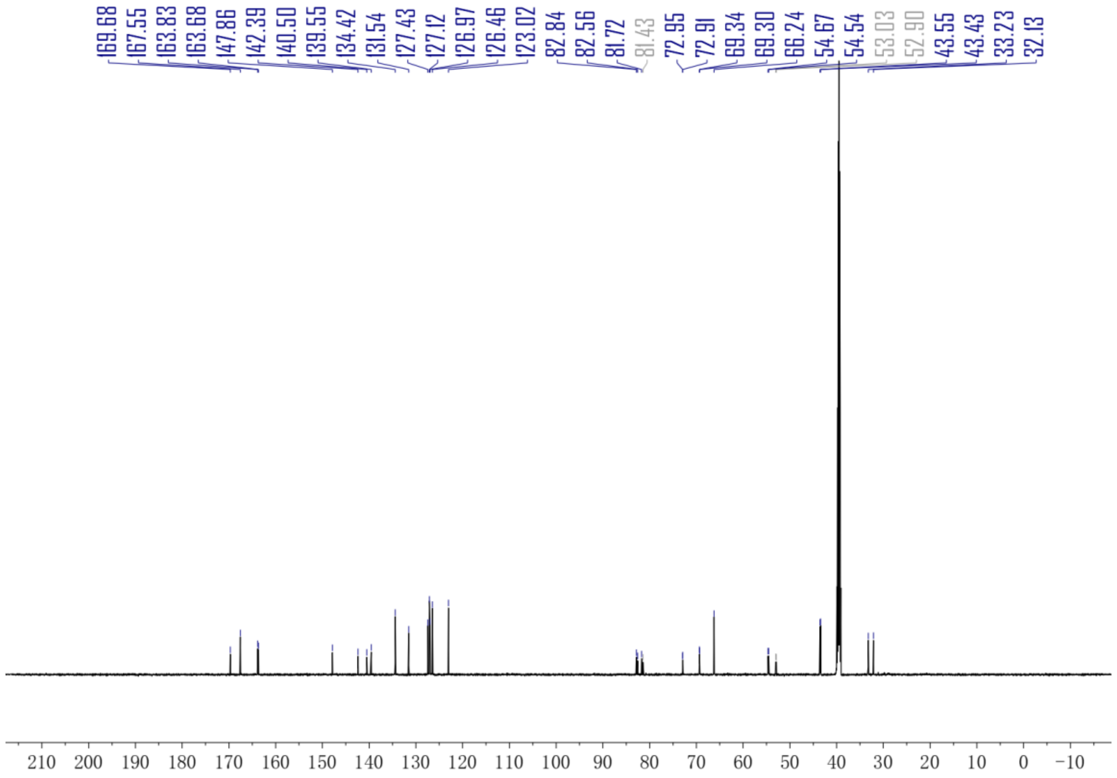

^{13}C-NMR spectrum of compound F14 (150 MHz, DMSO).

^{13}C NMR (150 MHz, DMSO) δ 169.68, 167.55, 163.83, 163.68, 147.86, 142.39, 140.50, 139.55, 134.42, 131.54, 127.43, 127.12, 126.97, 126.46, 123.02, 82.84, 82.56, 81.72, 81.43, 72.95, 72.91, 69.34, 69.30, 66.24, 54.67, 54.54, 53.03, 52.90, 43.55, 43.43, 33.23, 32.13.

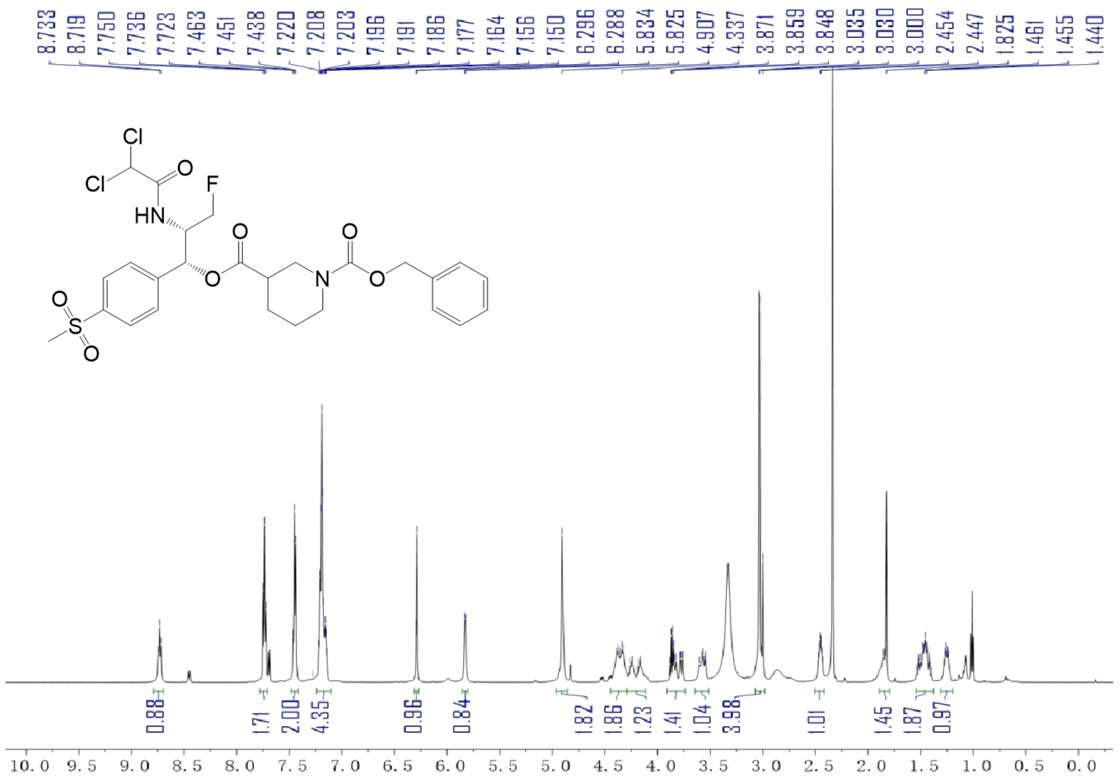

^{1}H-NMR spectrum of compound F15 (600 MHz, DMSO).

^{1}H NMR (600 MHz, DMSO-d_6) δ 8.73 (t, *J* = 8.0 Hz, 1H), 7.74 (t, *J* = 8.2 Hz, 2H), 7.44 (d, *J* = 7.8 Hz, 2H), 7.24 – 7.10 (m, 4H), 6.29 (d, *J* = 5.1 Hz, 1H), 5.83 (d, *J* = 5.3 Hz, 1H), 4.91 (s, 2H), 4.36 (d, *J* = 31.4 Hz, 2H), 4.20 (d, *J* = 46.6 Hz, 1H), 3.91 – 3.74 (m, 1H), 3.64 – 3.51 (m, 1H), 3.07 – 2.98 (m, 4H), 2.45 (dq, *J* = 9.8, 4.6, 4.2 Hz, 1H), 1.84 (d, *J* = 19.4 Hz, 1H), 1.47 (tdd, *J* = 25.3, 12.1, 5.5 Hz, 2H), 1.31 – 1.20 (m, 1H).

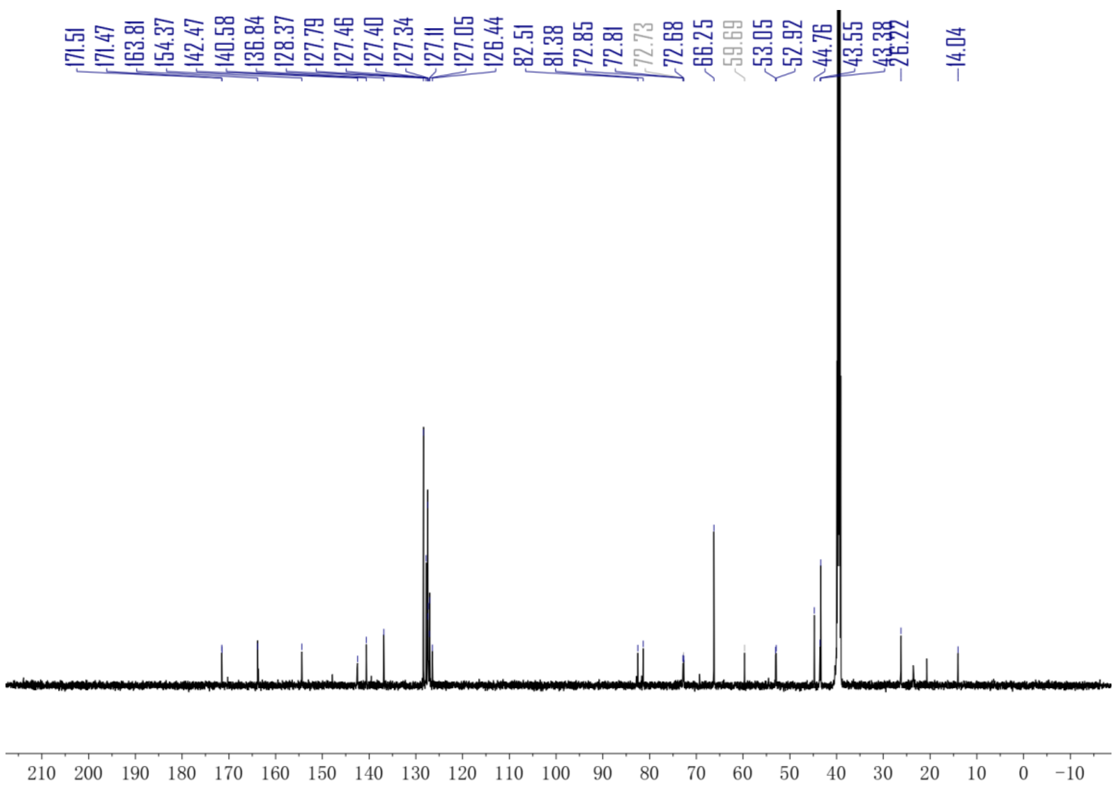

^{13}C-NMR spectrum of compound F15 (150 MHz, DMSO).

^{13}C NMR (150 MHz, DMSO) δ 171.51, 171.47, 163.81, 154.37, 142.47, 140.58, 136.84, 128.37, 127.79, 127.46, 127.40, 127.34, 127.11, 127.05, 126.44, 82.51, 81.38, 72.85, 72.81, 72.73, 72.68, 66.25, 59.69, 53.05, 52.92, 44.76, 43.55, 43.38, 26.22, 14.04.

Supplementary Note 4. Uncropped and unprocessed scans of SDS-PAGE

These include Fig. 5e (The expression of arcB was analysed by SDS-PAGE) and Supplemental Fig. 11a (The expression of arcA was analysed by SDS-PAGE).

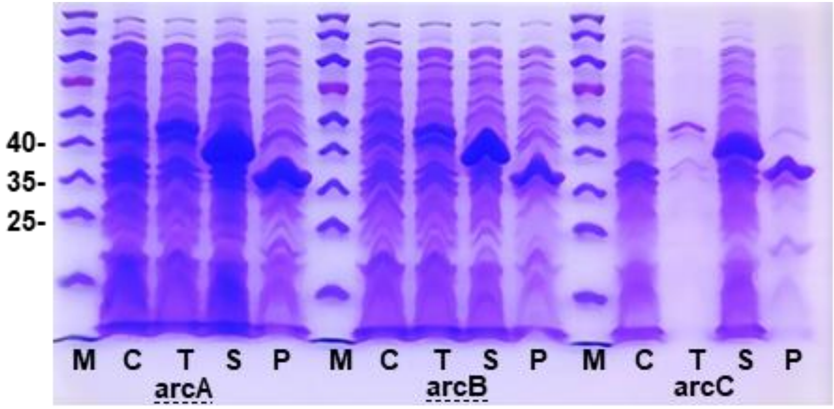

Supplement: Supplementary file 1 — Supplementary Information [file 41467_2024_50453_MOESM1_ESM.pdf]
