# Supplementary material for: A synthetic antibiotic class with a deeply-optimized design for overcoming bacterial resistance
Source: Nat Commun. 2024 Jul 18;15:6040. doi: 10.1038/s41467-024-50453-3 (PMC11255307; doi:10.1038/s41467-024-50453-3)
Supplement: Supplementary file 3 — Reporting Summary [file 41467_2024_50453_MOESM3_ESM.pdf]

nature portfolio

Corresponding author(s): Xu Wang
The field of biology

Last updated by author(s): Jun 10, 2024

Reporting Summary

Nature Portfolio wishes to improve the reproducibility of the work that we publish. This form provides structure for consistency and transparency in reporting. For further information on Nature Portfolio policies, see our Editorial Policies and the Editorial Policy Checklist.

Statistics

For all statistical analyses, confirm that the following items are present in the figure legend, table legend, main text, or Methods section.

n/a	Confirmed	
☐	☒	The exact sample size (*n*) for each experimental group/condition, given as a discrete number and unit of measurement
☐	☒	A statement on whether measurements were taken from distinct samples or whether the same sample was measured repeatedly
☐	☒	The statistical test(s) used AND whether they are one- or two-sided<br>*Only common tests should be described solely by name; describe more complex techniques in the Methods section.*
☐	☒	A description of all covariates tested
☐	☒	A description of any assumptions or corrections, such as tests of normality and adjustment for multiple comparisons
☐	☒	A full description of the statistical parameters including central tendency (e.g. means) or other basic estimates (e.g. regression coefficient) AND variation (e.g. standard deviation) or associated estimates of uncertainty (e.g. confidence intervals)
☐	☒	For null hypothesis testing, the test statistic (e.g. *F*, *t*, *r*) with confidence intervals, effect sizes, degrees of freedom and *P* value noted<br>*Give P values as exact values whenever suitable.*
☒	☐	For Bayesian analysis, information on the choice of priors and Markov chain Monte Carlo settings
☒	☐	For hierarchical and complex designs, identification of the appropriate level for tests and full reporting of outcomes
☒	☐	Estimates of effect sizes (e.g. Cohen's *d*, Pearson's *r*), indicating how they were calculated

Our web collection on statistics for biologists contains articles on many of the points above.

Software and code

Policy information about availability of computer code

Data collection	Infinite M200 Microplate reader; Nano Drop microspectrophotometer; RNA-Seq; CFX96 Real-Time PCR Assay System; MicroCal ITC; nanoDSF;
Data analysis	GraphPad Prism 8; Microsoft Office Excel 2010; All ITC experiments were carried out and analysed using Launch NanoAnalyze Software; All DSF experiments were performed using PR. ChemControl Software; The data (Transcriptomics, Metabolomics, Proteomics) were uploaded to the Majorbio cloud platform(https://cloud.majorbio.com) after the database search for data analysis; Scoring calculations were calculated by CScore in the SYBYL-X Suite.

For manuscripts utilizing custom algorithms or software that are central to the research but not yet described in published literature, software must be made available to editors and reviewers. We strongly encourage code deposition in a community repository (e.g. GitHub). See the Nature Portfolio guidelines for submitting code & software for further information.

Data

Policy information about availability of data

All manuscripts must include a data availability statement. This statement should provide the following information, where applicable:

- Accession codes, unique identifiers, or web links for publicly available datasets
- A description of any restrictions on data availability
- For clinical datasets or third party data, please ensure that the statement adheres to our policy

The data that support the findings of this study are available from the corresponding author on request. Source data are provided with this paper. The

transcriptomics data generated in this study have been deposited in the Genome Sequence Archive database under accession code subCRA027241 [https://ngdc.cncb.ac.cn/gsa/]. The metabolomics data generated in this study have been deposited in the OMIX, China National Center for Bioinformation / Beijing Institute of Genomics, Chinese Academy of Sciences, under accession code OMIX006632, OMIX006633 [https://ngdc.cncb.ac.cn/omix]. The proteomics data generated in this study have been deposited in the OMIX, China National Center for Bioinformation / Beijing Institute of Genomics, Chinese Academy of Sciences, under accession code OMIX006634, OMIX006635, OMIX006636 and OMIX006637 [https://ngdc.cncb.ac.cn/omix].

Research involving human participants, their data, or biological material

Policy information about studies with human participants or human data. See also policy information about sex, gender (identity/presentation), and sexual orientation and race, ethnicity and racism.

Reporting on sex and gender	N/A
Reporting on race, ethnicity, or other socially relevant groupings	N/A
Population characteristics	N/A
Recruitment	N/A
Ethics oversight	N/A

Note that full information on the approval of the study protocol must also be provided in the manuscript.

Field-specific reporting

Please select the one below that is the best fit for your research. If you are not sure, read the appropriate sections before making your selection.

☒ Life sciences ☐ Behavioural & social sciences ☐ Ecological, evolutionary & environmental sciences

For a reference copy of the document with all sections, see nature.com/documents/nr-reporting-summary-flat.pdf

Life sciences study design

All studies must disclose on these points even when the disclosure is negative.

Sample size	Experiments were performed with a minimum of three independent replicates (n=3-10, as indicated in the figure legends). The sample size was determined based on standard experimental designs in this field, without a pre-calculation of the sample size. A typical sample size of 3-10 was chosen to ensure sufficient statistical power and to minimize random variations.
Data exclusions	No data were excluded from the analyses.
Replication	Reproducibility was ensured by at least 3 independent replicates.
Randomization	All samples and animals are randomly divided into different experimental groups as indicated.
Blinding	For mouse study, investigators were blinded to group allocation during data collection and analysis. For other studies, experiments were performed blinded.

Reporting for specific materials, systems and methods

We require information from authors about some types of materials, experimental systems and methods used in many studies. Here, indicate whether each material, system or method listed is relevant to your study. If you are not sure if a list item applies to your research, read the appropriate section before selecting a response.

Materials & experimental systems

n/a	Involved in the study	
☐	☒	Antibodies
☐	☒	Eukaryotic cell lines
☒	☐	Palaeontology and archaeology
☐	☒	Animals and other organisms
☒	☐	Clinical data
☒	☐	Dual use research of concern
☒	☐	Plants

Methods

n/a	Involved in the study	
☒	☐	ChIP-seq
☒	☐	Flow cytometry
☒	☐	MRI-based neuroimaging

Antibodies

Antibodies used	anti-His tag antibody (AE003, ABclonal, China).
Validation	The fractions of pure product, total protein, supernatant, and precipitate were then analysed by SDS-PAGE and WB using an anti-His tag antibody (AE003, ABclonal, China). The antibody dilution ratio is 1:5000.

Eukaryotic cell lines

Policy information about cell lines and Sex and Gender in Research

Cell line source(s)	Vero, L0-2 and pk15 cells
Authentication	Vero, L02 and pk15 were obtained from China center for type culture collection (Wuhan University), National Microbial Resource Center. Catalog number of Vero: GDC0029. Catalog number of pk-15: GDC0061.
Mycoplasma contamination	All cell lines tested negative for mycoplasma contamination.
Commonly misidentified lines (See ICLAC register)	Not applicable.

Animals and other research organisms

Policy information about studies involving animals; ARRIVE guidelines recommended for reporting animal research, and Sex and Gender in Research

Laboratory animals	All experimental procedures were conducted in accordance with animal welfare guidelines, and were previously approved by the Animal Welfare and Ethics Committee of the Huazhong Agricultural University Wuhan, China (approval permit numbers: 202311010007 and 202311010008). All specific pathogen-free (SPF) Kunming mice (6–7 weeks old, weighing approximately 25 g) and SPF C57BL/6 mice (6–7 weeks old, weighing 18 ± 2 g) were purchased from Hubei Provincial Laboratory Animal Center (Wuhan, China). Animals were housed under standard humidity (50 ± 10%), temperature (25 ± 2?), and light-dark cycle (12 h each) conditions with free access to food and water.
Wild animals	No wild animals were used in the study.
Reporting on sex	Sex as a biological variable was not taken into consideration in this study.
Field-collected samples	No field-collected samples were used in the study.
Ethics oversight	Huazhong Agricultural University Wuhan, China

Note that full information on the approval of the study protocol must also be provided in the manuscript.

Plants

Seed stocks	N/A
Novel plant genotypes	N/A
Authentication	N/A
